# Heart and neural crest derivative 2‐induced preservation of sympathetic neurons attenuates sarcopenia with aging

**DOI:** 10.1002/jcsm.12644

**Published:** 2020-11-30

**Authors:** Anna Carolina Zaia Rodrigues, Zhong‐Min Wang, María Laura Messi, Henry Jacob Bonilla, Liang Liu, Willard M. Freeman, Osvaldo Delbono

**Affiliations:** ^1^ Department of Internal Medicine, Section on Gerontology and Geriatric Medicine Wake Forest School of Medicine Winston‐Salem NC USA; ^2^ Neuroscience Program Wake Forest School of Medicine Winston‐Salem NC USA; ^3^ Comprehensive Cancer Center Wake Forest School of Medicine Winston‐Salem NC USA; ^4^ Sticht Center for Healthy Aging and Alzheimer's Prevention Wake Forest School of Medicine Winston‐Salem NC USA; ^5^ Reynolds Oklahoma Center on Aging Oklahoma City OK USA

**Keywords:** Skeletal muscle, Neuromuscular junction, Denervation, Sarcopenia, Sympathetic nervous system, Aging

## Abstract

**Background:**

Sarcopenia, or age‐dependent decline in muscle force and power, impairs mobility, increasing the risk of falls, institutionalization, co‐morbidity, and premature death. The discovery of adrenoceptors, which mediate the effects of the sympathetic nervous system (SNS) neurotransmitter norepinephrine on specific tissues, sparked the development of sympathomimetics that have profound influence on skeletal muscle mass. However, chronic administration has serious side effects that preclude their use for muscle‐wasting conditions. Interventions that can adjust neurotransmitter release to changing physiological demands depend on understanding how the SNS affects neuromuscular transmission, muscle motor innervation, and muscle mass.

**Methods:**

We examined age‐dependent expression of the *heart and neural crest derivative 2* (Hand2), a critical transcription factor for SN maintenance, and we tested the possibility that inducing its expression exclusively in sympathetic neurons (SN) will prevent (i) motor denervation, (ii) impaired neuromuscular junction (NMJ) transmission, and (iii) loss of muscle mass and function in old mice. To test this hypothesis, we delivered a viral vector carrying Hand2 expression or an empty vector exclusively in SNs by vein injection in 16‐month‐old C57BL/6 mice that were sacrificed 6 months later. Techniques include RNA‐sequencing, real‐time PCR, genomic DNA methylation, viral vector construct, tissue immunohistochemistry, immunoblot, confocal microscopy, electrophysiology, and *in vivo* mouse physical performance.

**Results:**

Hand2 expression declines throughout life, but inducing its expression increased (i) the number and size of SNs, (ii) muscle sympathetic innervation, (iii) muscle weight and force and whole‐body strength, (iv) myofiber size but not muscle fibre‐type composition, (v) NMJ transmission and nerve‐evoked muscle force, and (vi) motor innervation in old mice. Additionally, the SN controls a set of genes to reduce inflammation and to promote transcription factor activity, cell signalling, and synapse in the skeletal muscle. Hand2 DNA methylation may contribute, at least partially, to gene silencing.

**Conclusions:**

Selective expression of Hand2 in the mouse SNs from middle age through old age increases muscle mass and force by (i) regulating skeletal muscle sympathetic and motor innervation; (ii) improving acetylcholine receptor stability and NMJ transmission; (iii) preventing inflammation and myofibrillar protein degradation; (iv) increasing autophagy; and (v) probably enhancing protein synthesis.

## Introduction

Sarcopenia, or age‐dependent decline in muscle force and power, impairs mobility, increasing the risk of falls, institutionalization, co‐morbidity, and premature death.^1^ Of several mechanisms proposed to explain its onset and progression, myofiber motor denervation is a primary culprit.^1^ We reported a higher percentage of partially and completely denervated fibres in aging mice than previously thought,^2^ and we defined the extent of skeletal muscle denervation in older adults^3^; however, the underlying mechanisms and, hence, therapeutic strategies to prevent or ameliorate the relentless advance of sarcopenia remain unknown. Whether muscle denervation starts at the myofiber or the central or peripheral nervous system is controversial,^4^, ^5^ and only recently have researchers explored whether motoneuron interaction with the cells that sustain innervation deteriorates over time. Answering these questions is essential for developing targeted interventions to prevent or reverse age‐related decline in skeletal muscle innervation and sarcopenia.

Another pathway involves the neuromuscular junction (NMJ); increasing evidence confirms diminished neural influence on skeletal muscle at older ages. Predominant denervation of fast‐twitch myofibers^6^ is followed by myofiber atrophy and motor unit remodeling^4^, ^7^, ^8^ that appears to favour reinnervation by slow motoneurons.^9^, ^10^, ^11^ Although we demonstrated the plasticity of muscle innervation in older adults who practiced an exercise regimen, the benefits were partial, variable, and difficult to sustain for most. Until we can target the mechanisms that drive NMJ instability and muscle denervation, we cannot develop effective interventions to prevent or reverse age‐related decline in skeletal muscle mass and force.^8^, ^12^, ^13^

In humans, symptoms of autonomic nervous failure arise with aging.^14^ Autonomic alterations and muscle weakness have been reported in several nervous system disorders and such age‐associated neurodegenerative diseases as amyotrophic lateral sclerosis (ALS), Parkinson's disease, and Alzheimer's disease.^15^, ^16^, ^17^, ^18^, ^19^ With normal aging, several changes in the autonomic nervous system may impair adaptation to common physiologic stressors and increase the risk of developing diseases that, in turn, harm autonomic function. Although this observation has been established for some cells and organ systems,^14^ the autonomic nervous system's long‐term role in stabilizing the NMJ has been reported only recently in animal models of systemic chemical or focal surgical ablation of the sympathetic nervous system (SNS).^20^, ^21^, ^22^

Whether SNS interventions can regulate aging NMJ structure and function and skeletal muscle motor innervation and mass is unknown. Sympathetic axons innervate skeletal muscle fibres^23^, ^24^ at the NMJ and extrasynaptic sarcolemma,^20^, ^25^ but research is only now exploring their role in maintaining skeletal muscle structure and function.^20^, ^21^, ^26^, ^27^ Investigators using loss‐of‐function approaches have learned that the SNS regulates motor nerve synaptic vesicle release, the skeletal muscle transcriptome, muscle force generated by motor nerve activity, axonal neurofilament phosphorylation, myelin thickness, and myofiber subtype composition and cross‐sectional area (CSA).^20^, ^21^, ^26^ It also regulates the guanine nucleotide‐binding protein Gα_i2_ pathway to modulate post‐synaptic membrane acetylcholine (ACh) receptor (AChR) levels.^20^

We propose that loss of sympathetic neuron (SN) function destabilizes motor innervation, leading to sarcopenia. Recently, we found that noradrenaline (NA) discharge at SN terminals modulates motoneurons' spontaneous and evoked release of synaptic vesicle ACh through the coordinated activity of transient receptor potential cation channels (TRPV) and β1‐AR and α2B‐AR.^26^ A comprehensive approach enabled us to examine the influence of the SNS on muscle motor innervation: it includes nerve immunolabelling co‐registration and electrophysiological recordings in a novel mouse *ex vivo* preparation that preserves the complexity of the interaction between motoneurons and the SNS at the NMJ and muscle innervation in living mice. We found blunted SN regulation of motoneuron synaptic vesicle release, mediated by β1‐AR and α2B‐AR, in geriatric mice. We propose that age‐associated sympathetic hyperactivity explains AR down‐regulation at the NMJ presynapse but have yet to determine whether a decrease in SNs also accounts for the decrease in AR expression.

If sympathomimetic agents enhance NMJ transmission and remediate muscle wasting and strength in aged rodents,^28^ why do endogenously secreted catecholamines fail to maintain muscle mass and force in the absence of significant changes in β2‐AR levels? Recent studies support a novel mechanism by which motor and SNs interact at the NMJ presynapse.^20^, ^21^, ^26^ Impaired neuronal cross‐talk can explain, at least partially, progressive atrophy and weakness with aging.

Maintaining or recovering sympathetic muscle innervation throughout life is undoubtedly preferable to chronic use of AR pharmacological agents. Drug administration cannot mimic the complex, concerted tissue response to endogenous SN neurotransmitters, which depends on the AR subtype's repertoire and location. Loss of receptor selectivity, tolerance, and tachyphylaxis can also trigger adverse effects. Moreover, pharmaceutical compounds reach stable levels in blood, precluding any adaptation to physiological or pathological challenges.

Based on these observations, we sought a new approach to prevent sarcopenia. We examined age‐dependent expression of the *heart and neural crest derivative 2* (Hand2), a critical transcription factor for post‐mitotic maintenance of SNs,^29^, ^30^ and we tested the possibility that inducing its expression exclusively in SNs will significantly prevent (i) motor denervation, (ii) impaired NMJ transmission, and (iii) loss of muscle mass and function in old mice.

## Materials and methods

### Animals and ethics statement

Middle‐aged (16‐month‐old) male and female C57BL/6 mice were obtained from the National Institute on Aging and housed in the pathogen‐free Animal Research Program of the Wake Forest School of Medicine (WFSM). After viral vector injections, they were maintained at 21°C and a 12:12 h dark–light cycle for 6 months and euthanized at 22 months. All mice were fed chow *ad libitum* and had continuous access to drinking water. All experimental procedures were conducted in compliance with National Institutes of Health (NIH) laboratory animal care guidelines. We made every effort to minimize suffering. Protocols A15‐219 and A‐18‐204 were approved by the WFSM Institutional Animal Care and Use Committee for this study.

### RNA‐Seq and data analysis

Stranded RNA‐Seq (TruSeq v2, Illumina) was performed on 500 ng of total RNA from each sample [empty vector (EV), *n* = 10, Hand2, *n* = 7]. Following sequencing, reads were trimmed and aligned, and differential expression statistics and correlation analyses were performed in Strand NGS software package (Agilent). Reads were aligned against the Mm10 build of the mouse genome (2014.11.26). For technical details, see the Supporting Information.

### Genomic *heart and neural crest derivative 2* DNA methylation

Base‐specific quantitation of *Hand2* methylation was performed by Bisulfite Amplicon Sequencing. PCR primers were designed to amplify bisulfite‐converted DNA regions of the *Hand 2* promoter and gene body, with two regions passing quality control. Sample DNA was bisulfite converted, and PCR amplicons were generated from each sample. Amplicons from each sample were pooled, and Nextera XT library preparation was performed. Libraries were sequencing on an Illumina MiSeq. For data analysis, we used CLC workbench workflow and Illumina BaseSpace according to standard methods. Methylation levels of each cytosine were determined in CG, CH, or CHH contexts in region 1 (57 320 498–57 321 005 chromosome 8) and region 2 (57 321 202–57 322 499 chromosome 8) by subtracting the unconverted read counts from the total read counts for that site.^31^

### Real‐time quantitative PCR

Messenger RNA‐expression levels were quantified by qPCR using a SensiFAST Probe Lo‐ROX One‐Step Kit (Bioline, Taunton, MA). Total RNA (20 ng) was loaded for qPCR in 20 μL total volume. After real‐time (RT) cDNA synthesis at 45°C for 30 min and RT deactivation at 95°C for 5 min, PCR was performed over 40 cycles at 95°C for 10 s and at 60°C for 1 min. For data normalization and analysis, see the Supporting Information.

For viral vector constructs and delivery, skeletal muscle immunofluorescence analyses, whole‐mounted lumbricalis muscle immunohistochemistry, and paravertebral sympathetic ganglion confocal microscopy imaging, see the Supporting Information.

### Neurofilament or TH+ path length and neuromuscular junction co‐localization analysis in confocal images

Post‐terminal fragments and pre‐terminal and post‐terminal co‐localization were quantified using Fiji‐ImageJ software (NIH) particle analysis and an EZ co‐localization plugin, respectively. Images were converted to 8‐bit type and thresholded for particle analysis, while co‐localization was performed on maximal intensity stack projections. Phosphorylated (detected with SMI 312 Ab) or nonphosphorylated (SMI 311 Ab) neurofilament or TH+ path length was quantified on flattened images using the Simple Neurite Tracer plugin. Exclusion of structures with a diameter larger than 3.5 μm excluded blood vessels from the analysis.

For electrophysiological recording of neuromuscular transmission, assessment of muscle force generated by direct muscle‐evoked or nerve‐evoked stimulation, protein isolation and immunoblots, *in vivo* fatigue recording, inverted‐cling grip test, and spontaneous locomotor activity, see the Supporting Information.

### Statistical analysis

All experiments and analyses were conducted blind to treatment group. No statistical methods were used to predetermine sample sizes; however, our sample sizes are similar to those reported in recently published studies.^32^, ^33^ Sigma Plot version 12.5 (Systat Software, Inc., San Jose, CA) and Microsoft Excel software were used for statistical analyses. All data were expressed as mean ± SEM. Student's *t*‐test was used to compare two groups, and analysis of variance (ANOVA) followed by Bonferroni's post‐hoc analysis was used to compare three or more. An ANOVA repeated‐measures test was used to compare means across one or more variables that were based on repeated observations. The figure legends indicate the specific tests for each set of experiments. A *P*‐value < 0.05 was considered significant.

## Results

**FIGURE 1:**
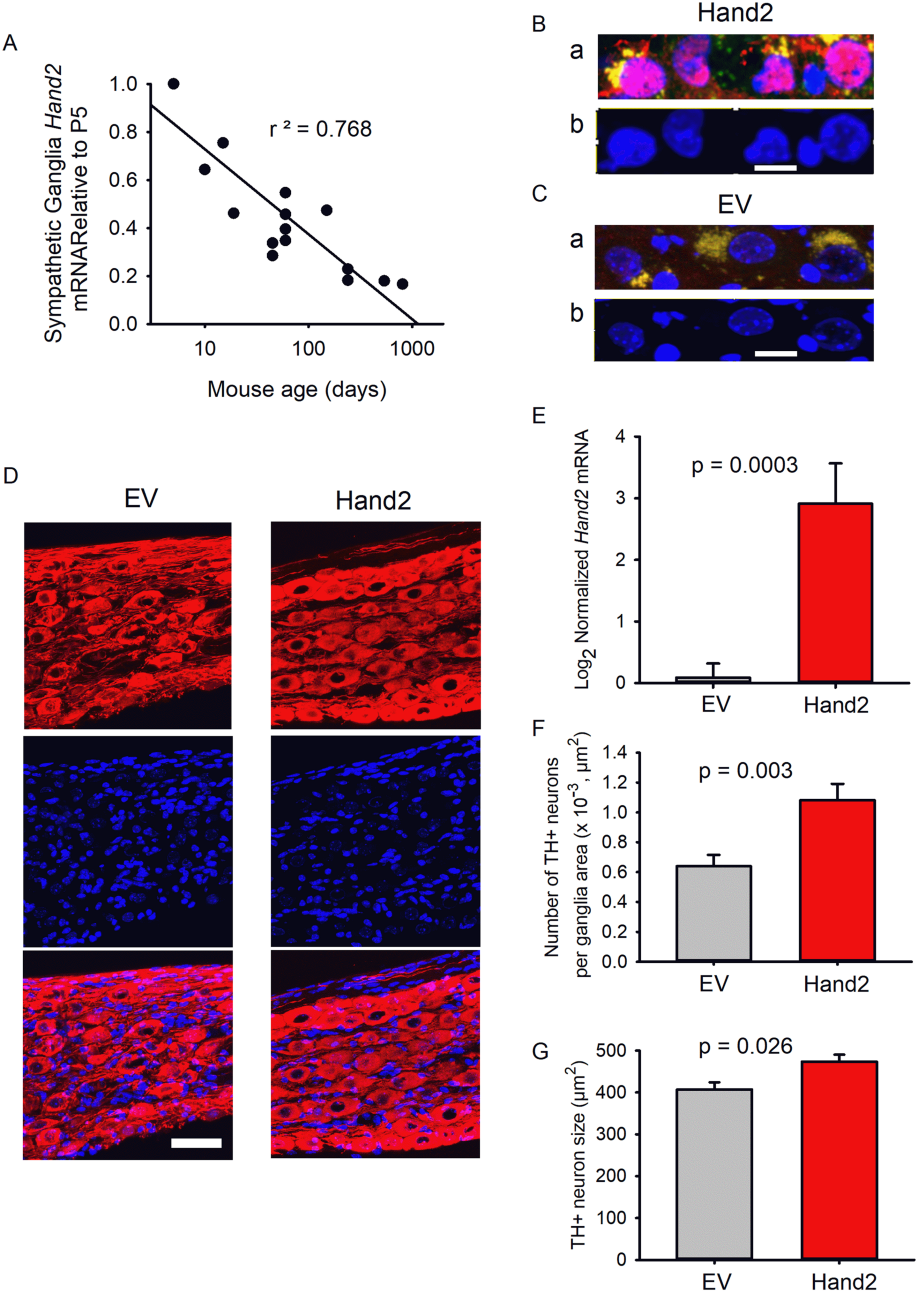
Restoration of paravertebral sympathetic ganglion heart and neural crest derivative 2 (*Hand2*) mRNA preserves the number and size of sympathetic neurons lost with aging. *(A) Hand2* mRNA levels relative to post‐natal Day 5 (P5) were measured by quantitative real‐time PCR (qPCR). Data represent the mean of three ganglia from three different mice per point. Their ages ranged from 5 days to 27 months. Linear regression shows an inverse correlation between mouse age and sympathetic neuron *Hand2* mRNA. *(Ba)* Sympathetic ganglion neurons from Hand2‐treated mice show Hand2 protein immunoreactivity to the Hand2 Ab (red); nuclei staining with Hoechst 33342 (blue); and perinuclear EGFP fluorescence (green). The nuclear Hand2 and DNA overlay appears pink. *(Bb)* Hand2‐positive neurons display relaxed chromatin. *(Ca)* ganglion neurons from empty vector (EV)‐treated mice do not show Hand2 protein immunoreactivity to the Hand2 Ab, while their nuclei are positive to staining with Hoechst 33342 (blue), and the perinuclear area shows EGFP expression (green). *(Cb)* Neurons expressing EGFP exhibit relaxed chromatin. Calibration bars in *(Bb)* and *(Cb)*, 30 μm. *(D)* EV‐treated and Hand‐treated mice show TH+ neurons (red), EGFP expression (green), nucleus staining (blue), and overlay of the three channels (bottom row) in confocal z‐stack images. Calibration bar = 50 μm. Compared with those from the EV group, ganglion neurons from the Hand2‐treated mice show significantly higher *Hand2* mRNA levels (*n* = 10 ganglia from 10 EV‐treated mice and*n* = 7 ganglia from 7 Hand2‐treated mice)*(E)*, more TH+ neurons (*n* = 7 ganglion confocal images from 5 EV‐treated mice and *n* = 7 ganglion confocal images from 5 Hand2‐treated mice) *(F)*, and larger TH+ neurons (*n* = 7 ganglion confocal images from 5 EV‐treated mice and *n* = 7 ganglion images from 5 Hand2‐treated mice) *(G)*. Data in *(E–G)* were statistically analysed by nonpaired *t*‐test.

In mice, Hand2 expression declines throughout life, but inducing its expression preserves the number and size of paravertebral sympathetic ganglion neurons. *Figure* 1A shows that *Hand2* mRNA declines over time; its levels drop >50% from post‐natal Day 5 (P5) to 16 months of age. To determine whether sustaining Hand2 levels in SNs increases their number and enlarges their soma area, we systemically delivered a viral vector carrying Hand2 expression (ITR‐CAG‐EGFP‐PRSX8‐Hand2‐WPRE‐ITR‐serotype 9) or an EV (ITR‐CAG‐EGFP‐PRSX8‐WPRE‐ITR‐serotype 9) by saphenous vein injection in 16‐month‐old mice that were sacrificed 6 months later.

Paravertebral sympathetic ganglion neurons from mice treated with Hand2, but not EV, show immunoreactivity to a Hand2 antibody (*Figure* 1BBa, b). The perinuclear location of EGFP fluorescence (green), probably at the ER‐Golgi apparatus, is due to its expression separated from Hand2 (see Materials and Methods). *Figure* 1Bb shows that in 22‐month‐old mice, chromatin is relaxed in neurons expressing the Hand2 protein. In contrast, ganglia from the EV‐treated mice display EGFP fluorescence but not Hand2 (*Figure* 1Ca, b). The detection of Hand2 protein is consistent with the very significant increase in *Hand2* mRNA levels in the Hand2‐treated compared with EV‐treated mice (*Figure* 1E).

Confocal images of ganglion neurons from both groups (*Figure* 1D) show that they are tyrosine‐hydroxylase positive (TH+; red, top row) with nucleus staining (blue, middle row) and overlay of the two channels (bottom row). However, the Hand2‐treated mice have more ganglion neurons (*Figure* 1F) with larger soma size (*Figure* 1G) than the controls. These results indicate that sustained Hand2 expression in paravertebral SNs prevents their loss and atrophy in old mice.

### Increased heart and neural crest derivative 2 DNA methylation in paravertebral sympathetic ganglion neurons with aging may contribute to gene silencing

Sympathetic ganglion neurons from young (3–5 months), middle‐aged (13 months), and old (22 months) mice were collected to examine DNA methylation of CpG dinucleotides.^34^ After bisulfite treatment, we amplified genomic DNA using designed primers and performed Bisulfite Amplicon Sequencing analysis.^34^ *Figure* S1A and C shows that the Hand2 gene is methylated more in the old than the young and middle‐aged mice in both region 1 (Supplementary Database 1_Genomic DNA methylation_Region 1) and region 2 (Supplementary Database 2_Genomic DNA methylation_Region 2). Also, methylation takes place at CG but not C combined with any other nucleotide (CH or CHH) (B, D). Thus, hypermethylation may account, at least partially, for Hand2 gene silencing with aging.

### Sympathetic neuron heart and neural crest derivative 2 increases muscle weight and force and whole‐body strength in old mice

*Figure* S2 shows that compared with EV, Hand2 treatment significantly increased fast [tibialis anterior (TA) and extensor digitorum longus (EDL) muscle] (A, C), mixed‐fibre‐type [gastrocnemius (GA)] (B), and slow (soleus) (D) muscle weight. Body (E), heart (F), and visceral fat (G) weight did not differ significantly between treatment groups. Additionally, spontaneous mobility—tested as spontaneous maximum speed (H), average speed (I), total travelled distance (J), and time spent in motion (J) in an open arena setting—did not show statistically significant differences. However, compared with EV treatment, Hand2 treatment significantly enhanced net‐hanging time, a measurement of muscle strength (L), but treadmill running time did not differ between groups, despite Hand2's tendency to improve endurance (M). Running time for both groups was normalized to the maximal value, recorded for Hand2‐treated mice at 10 m/min. These results indicate that sustained Hand2 expression in SNs increases skeletal muscle weight and force and whole‐body strength in old mice.

### Preserved sympathetic neuron heart and neural crest derivative 2 expression increases myofiber size but does not modify muscle fibre‐type composition

**FIGURE 2:**
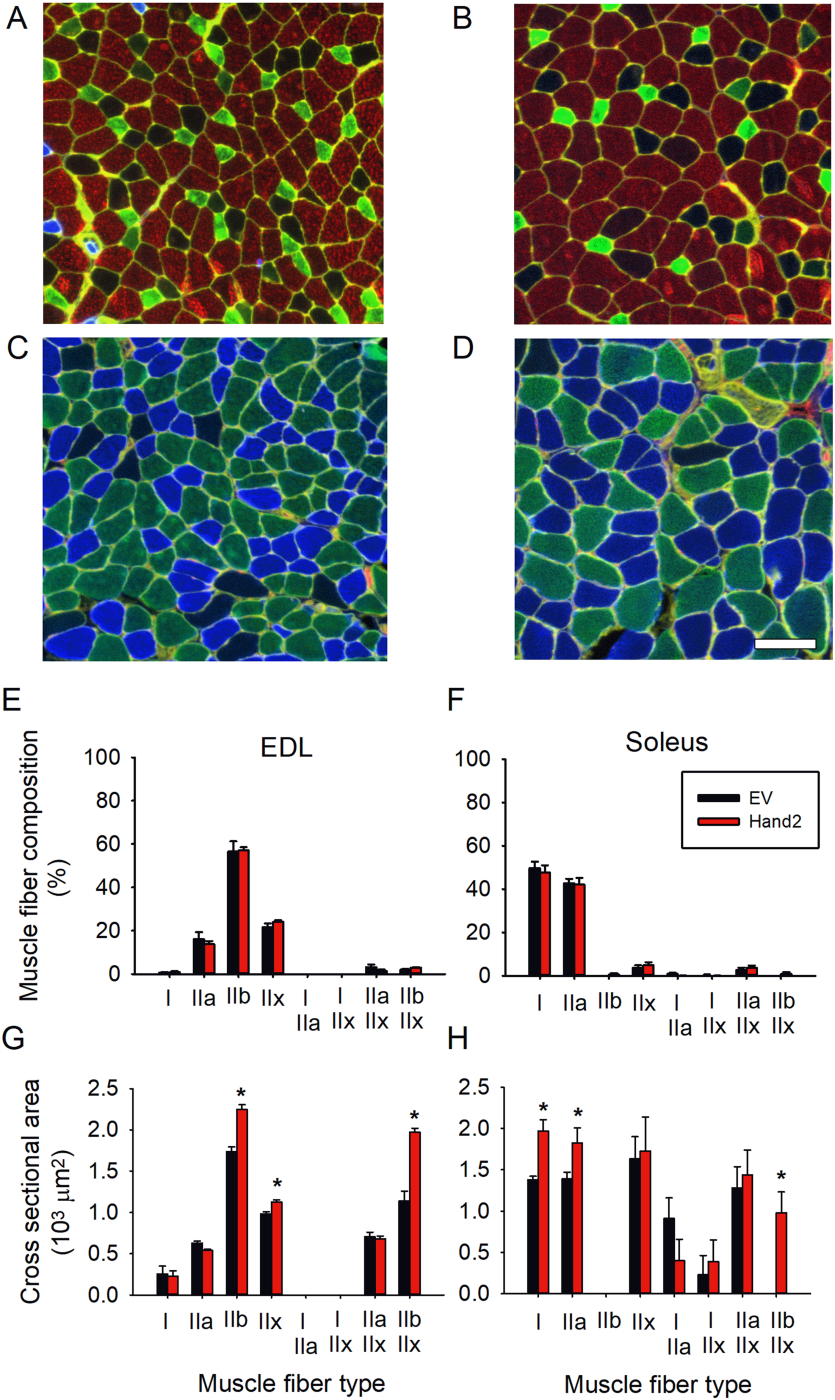
Heart and neural crest derivative 2 (Hand2) expression in sympathetic neurons attenuates age‐dependent muscle atrophy as assessed by myosin heavy chain (MHC) immunofluorescence. *(A, B)* Compared with empty vector (EV)‐treated mice *(A, C)*, Hand2‐treated mice show larger extensor digitorum longus (EDL) muscle *(B)* and soleus muscle *(D)* cross sections. Calibration bar = 50 μm. EDL *(E)* and soleus *(F)* pure and hybrid muscle fibre composition, expressed as a per cent of the total number of fibres, did not differ significantly between groups. In contrast, muscle fibre cross‐sectional area showed statistically significant between‐group differences in EDL for type IIb (*P* = 2.8E−05), IIx (1.7E−04), and IIb + IIx (2.3E−05) fibres and soleus for type I (*P* = 1.8E−03), IIa (0.05), and IIb + IIx (2.3E−03) fibres. *n* = 7 EDL or soleus muscles from 7 mice per group. Data were compared using one‐way ANOVA.

To determine whether increased lean mass accounts for the improved muscle weight and force, we quantified myofiber number and CSA in muscle transversal cuts stained with antibodies specific for myosin heavy chain subtypes. We selected EDL and soleus muscles for these determinations because of their highly predominant type II and type I fibre composition, respectively, and total muscle CSA, which is amenable to full quantification. *Figure* 2 shows that muscle fibre‐type composition did not differ with treatment (A–F), but some fibres' CSA increased in both muscles (A–D, G, H). Specifically, the CSA of EDL type IIb, IIx, and IIb/IIx (G) and soleus type I, IIa, and IIb/IIx (H) fibres increased significantly in Hand2‐treated but not EV‐treated mice. These results indicate that preserving sympathetic innervation significantly prevents age‐related muscle atrophy.

### Sustained heart and neural crest derivative 2 expression in sympathetic neurons enhances neuromuscular junction transmission and prevents loss of nerve‐evoked muscle force

**FIGURE 3:**
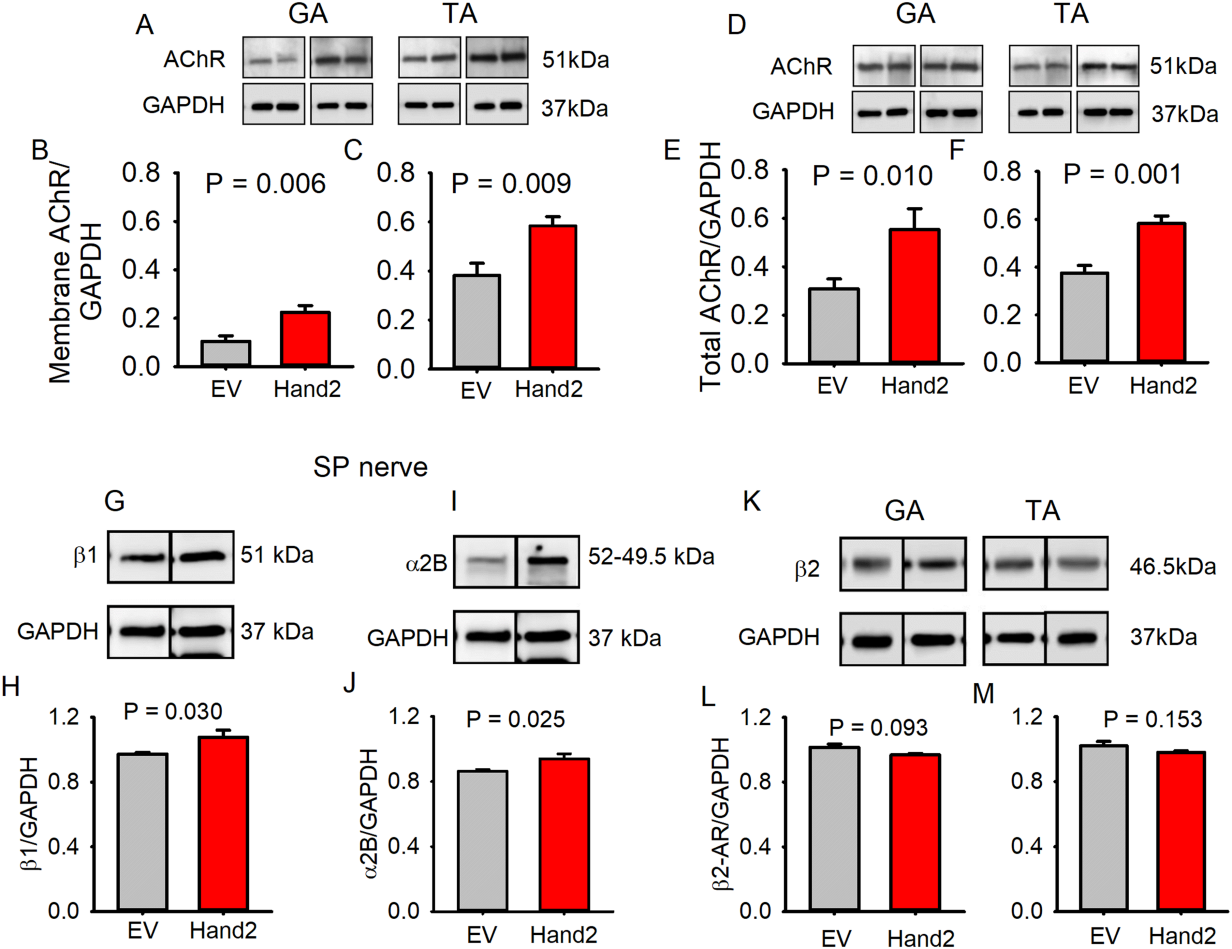
Sustained heart and neural crest derivative 2 (Hand2) expression in sympathetic neurons attenuates declines in acetylcholine receptor (AChR) levels and presynaptic β1 and α2b adrenergic receptors with aging. Immunoblot analysis of membrane *(A)* and total *(D)* AChR in gastrocnemius (GA) and tibialis anterior (TA) muscles from empty vector (EV)‐treated (*n* = 6 GA and*n* = 6 TA muscles from six different mice) and Hand2‐treated (*n* = 7 GA and *n* = 7 TA muscles from seven different mice) mice. The digital optical density of the bands was normalized to GAPDH and is quantified in graphs *(B, C)* and *(E, F)*. With the use of a similar approach, sciatic‐peroneal (SP) β1‐AR *(G, H)* and α2b‐AR *(I, J)* increased, but muscle β2 *(Κ–Μ)* showed no significant changes between groups with aging. *n* = 6 nerves or muscles from an equal number of mice per treatment group.

*Figure* S3 and *Table* 1 show increases in end‐plate potential (EPP) amplitude, recorded at 2, 10, 25, 30, 50, 100, and 150 Hz; quantal content; and spontaneous miniature EPP (MEPP) frequency and time to peak in the peroneal‐lumbricalis preparation.^21^, ^26^ These results indicate that preserving Hand2 levels in the SNs of aging mice enhances spontaneous and evoked neuromuscular transmission. To investigate whether preserving muscle sympathetic innervation stabilizes AChR and regulates pre‐terminal adrenergic receptor levels, we measured both groups of receptors by immunoblot. *Figure* 3 shows that Hand2 enhances both membrane (A–C) and total (D–F) AChR levels in TA and GA muscles as well as adrenergic receptor levels in presynaptic β1 (G, H) and α2B (I, J) but not β2 (K–M).

**TABLE 1:** Miniature end‐plate potentials (MEPPs) and end‐plate potentials (EPPs) recorded in the peroneal‐lumbricalis preparation from heart and neural crest derivative 2 (Hand2)‐treated and empty vector (EV)‐treated mice

	EV (46 fibres; 4 mice)		Hand2 (33 fibres; 3 mice)		
	Mean	SEM	Mean	SEM	*P*‐value
*MEPP*					
Amplitude (mV)	1.390	±0.050	1.320	±0.047	0.381
Frequency (Hz)	0.930	±0.105	1.297	±0.132	**0.031**
Time to peak (ms)	1.171	±0.078	0.829	±0.069	**0.007**
Half‐decay time (ms)	1.680	±0.104	1.901	±0.102	0.185
Duration (ms)	5.773	±0.391	6.275	±0.532	0.456
*EPP*					
Amplitude (mV)	19.937	±1.161	24.053	±1.579	**0.035**
Latency (ms)	1.974	±0.102	2.033	±0.113	0.700
Time to peak (ms)	3.325	±0.129	3.026	±0.156	0.142
Half‐decay time (ms)	6.529	±0.269	6.245	±0.321	0.500
Quantal content	15.381	±0.902	18.686	±1.303	**0.034**

*P* values in bold indicate statistically significant differences.

The number of fibres and mice examined per group are between parentheses.

**FIGURE 4:**
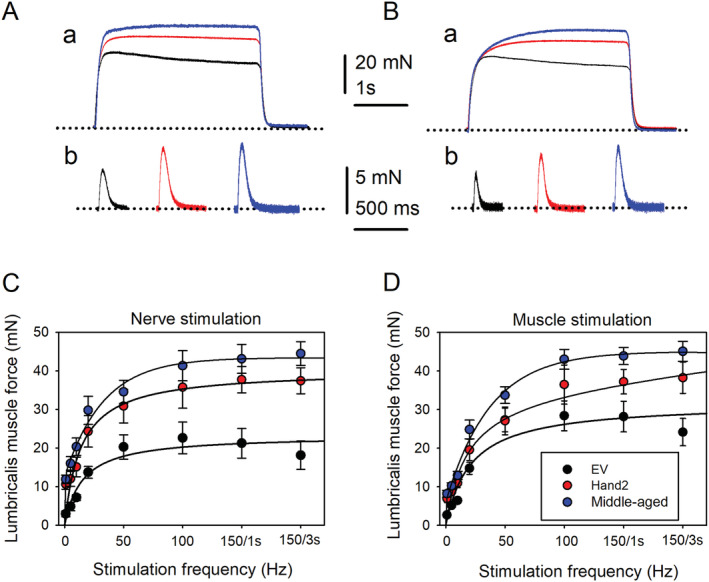
Heart and neural crest derivative 2 (Hand2) treatment preserves muscle force in response to single or repetitive nerve or muscle stimulation in old mice. *(Aa)* Tetanic force developed in response to 150 Hz pulses applied for 3 s to the distal peroneal nerve near the lumbricalis muscle from an untreated middle‐aged (blue), Hand2‐treated (red) or empty vector (EV)‐treated (black) mouse. *(Ab)* Single twitches recorded in the muscles tested in*(Aa)*. Dotted lines represent the baselines. *(Ba)* Tetanic force developed in response to 150 Hz pulses applied for 3 s to the lumbricalis muscle from a middle‐aged (blue), Hand2‐treated (red), or EV‐treated (black) mouse. *(Bb)* Single twitches recorded in the muscles tested in *(Ba)*. *(C)* Lumbricalis muscle force in millinewtons (mN) as a function of distal peroneal stimulation at increasing frequency (1–150 Hz). Differences between Hand2‐treated (red symbols; *n* = 8 muscles from eight mice) and EV‐treated (black symbols; *n* = 6 muscles from six mice) mice at 1, 5, 10, 20, 50, 100, 150/1 s, and 150/3 s are (mean ± SEM): 0.001, 0.022, 0.036, 0.038, 0.05, 0.05, 0.005, and 0.002, respectively. Differences between Hand2‐treated mice and middle‐aged mice (*n* = 8 muscles from 8 mice) were (*P*‐value): 0.143, 0.059, 0.055, 0.113, 0.164, 0.141, 0.113, and 0.058, at 1, 5, 10, 20, 50, 100, 150/1 s, and 150/3 s Hz, respectively. D. Lumbricalis muscle force as a function of direct stimulation at increasing frequencies. *n* = 6 muscles from 6 EV‐treated and 8 muscles from 8 Hand2‐treated mice. Differences between Hand2‐treated and EV‐treated mice at 1, 5, 10, 20, 50, 100, 150/1 s, and 150/3 s frequencies were (mean ± SEM): 0.005, 0.007, 0.003, 0.114, 0.482, 0.141, 0.05, and 0.02, respectively. Differences between Hand2‐treated mice (*n* = 8 muscles from eight mice) and middle‐aged mice (*n* = 8 muscles from eight mice) were *P*‐value = 0.245, 0.143, 0.137, 0.231, 0.269, 0.233, 0.089, and 0.183, at 1, 5, 10, 20, 50, 100, 150/1 s, and 150/3 s Hz, respectively. Data in *(C)* and *(D)* were statistically analysed using ANOVA repeated‐measures tests.

To determine whether improved neuromuscular transmission affects the generation of muscle force, we measured nerve‐evoked muscle contraction at increasing frequencies (2–150 Hz) in EV‐treated and Hand2‐treated mice using the same preparation.^21^ We elicited lumbricalis muscle contraction by plantar nerve stimulation and then blocked neuromuscular transmission with 10^−5^ g/mL d‐tubocurarine. After verifying that nerve stimulation did not evoke muscle contraction, we switched to direct muscle stimulation, applying field pulses and repeat maximal specific sub‐tetanic and tetanic force at the same frequencies. *Figure* 4Aa shows that the tetanic force in response to 150 Hz pulses applied for 3 s to the distal peroneal nerve near the lumbricalis muscle was not sustained and reached a lower peak in EV‐treated (black trace) than Hand2‐treated (red trace) mice. Single twitches recorded in these muscles also showed less force in the EV group (*Figure* 4Ab). Differences between the groups were significant at all frequencies (*Figure* 4C).

Tetanic force developed in response to direct stimulation of lumbricalis muscle was also not sustained and had a lower peak and weaker twitches in EV‐treated than Hand2‐treated mice (*Figure* 4Ba, b). However, for this effect, differences between groups were not significant at 20, 50, and 100 Hz (*Figure* 4D). Lumbricalis muscles from untreated middle‐aged mice (blue traces) developed more force that Hand2‐treated mice, but this difference did not reach statistical significance. In contrast, differences between middle‐aged and EV‐treated old mice were highly significant at all stimulation frequencies (*Figure* 4).

These results indicate that sustained Hand2 expression in SNs preserves NMJ transmission, muscle innervation, and muscle‐evoked and nerve‐evoked muscle contraction.

### Heart and neural crest derivative 2 attenuates neuromuscular junction post‐terminal fragmentation and denervation

*Figure* S4 compares muscle areas of the same dimensions enriched in NMJs. The EV‐treated mice (*Figure* S4A, C) obviously have more and smaller post‐terminals than the Hand2‐treated mice (*Figure* S4B, D). Histograms show that almost all post‐terminal events in EV muscle are smaller than 400 μm^2^ (*Figure* S4C), while Hand2 post‐terminal areas vary widely, some exceeding 1400 μm^2^ (*Figure* S4D). The EV group has five‐fold more post‐terminal fragments than the Hand2 group after normalizing their number to muscle area (*Figure* S4E).

**FIGURE 5:**
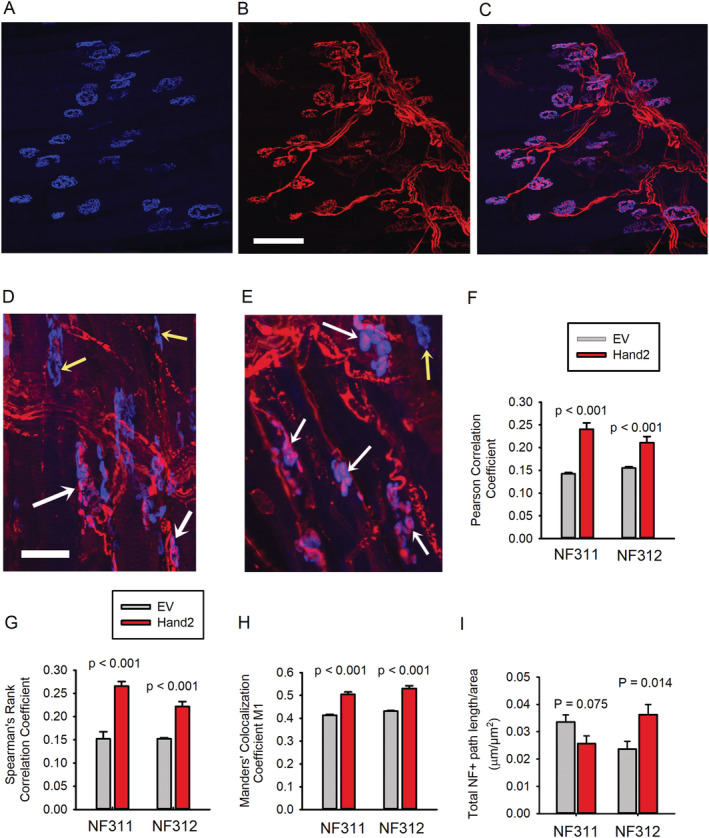
Sympathetic neuron heart and neural crest derivative 2 (Hand2) attenuateslumbricalis muscle fibre motor denervation with aging. *(A–C)* Confocal z‐stack images of neuromuscular junction (NMJs) from a young (4‐month‐old) mouse. The post‐terminal (blue) was stained with BGT‐680 *(A)*, the pre‐terminal immunostained with the SMI‐311 Ab against neurofilament (red) *(B)*, and *(C)*shows their overlay. *(D, E)* Pre‐terminal and post‐terminal NMJ co‐localization in pink (white arrows) and lack of overlay in blue (yellow arrows) in muscles from empty vector (EV)‐treated and Hand2‐treated mice, respectively. Calibration = 50 μm. *(F–H)* Pre‐terminal and post‐terminal NMJ co‐localization in muscles from EV‐treated or Hand2‐treated mice analysed by Pearson correlation coefficient *(F)*, Spearman's rank correlation coefficient *(G)*, and Manders' co‐localization coefficient M1 *(H)*. *n* = 10 muscles per treatment (five immunostained with SMI 311 and five with SMI 312 antibodies). *(I)* Total and phosphorylated NF paths per muscle area.

Next, we investigated whether SN Hand2 attenuates the muscle denervation seen with aging. As studies of mouse lumbricalis muscle innervation and NMJ transmission are recent,^21^, ^26^, ^27^ little is known about its myofiber innervation. *Figure* 5 shows a lumbricalis muscle from a 3‐month‐old mouse: 32 post‐terminals stained with labelled α‐bungarotoxin 680 nm (blue, A); pre‐terminals immunostained with neurofilaments S311 and SV2 (red, B); and their overlay (C). All post‐terminals seem to be innervated. Analysis of an average of 50 NMJs per muscle in six lumbricalis muscles from 3‐ to 4‐month‐old mice shows that 97 ± 1.5% are innervated. Thus, the lumbricalis, like other, more studied mouse muscles, such as the EDL and soleus, does not exhibit denervation in young mice. In contrast, the old mice show a mixture of innervated and denervated terminals, but in those treated with Hand2, more are innervated (white arrows) and fewer denervated (yellow arrows) than in the EV group (*Figure* 5D, E). To quantify the difference, we detected NMJ innervation with antibodies against either nonphosphorylated (SMI 311) or phosphorylated (SMI 312) neurofilament and analysed pre‐terminal and post‐terminal NMJ co‐localization using the Pearson correlation coefficient (*Figure* 5F), Spearman's rank correlation coefficient (*Figure* 5G), and Manders' co‐localization coefficient M1 (*Figure* 5H). The analysis shows that Hand2‐treated mice preserve innervation significantly more than the EV group.

To further investigate the mechanism underlying differences in NMJ innervation, we measured the total NF+ path length normalized to muscle area. The difference was not significant for nonphosphorylated NF but significant for phosphorylated NF (*Figure* 5I). In the Hand2‐treated mice, phosphorylation of the three NF subunits—heavy (NFH, *Figure* S5A), medium (NFM, *Figure* S5B), and light (NFL, *Figure* S5C)—was greater than in the EV‐treated mice, consistent with the analysis of NMJ innervation (*Figure* 5) and possibly explained by the significant decrease in phosphatases PP2A (*Figure* S5D) and PP1 (*Figure* S5E). These results indicate that sustained Hand2 expression in SNs attenuates NMJ post‐terminal fragmentation, myofiber denervation, and neurofilament dephosphorylation with aging.

### Heart and neural crest derivative 2 increases muscle sympathetic innervation with aging

**FIGURE 6:**
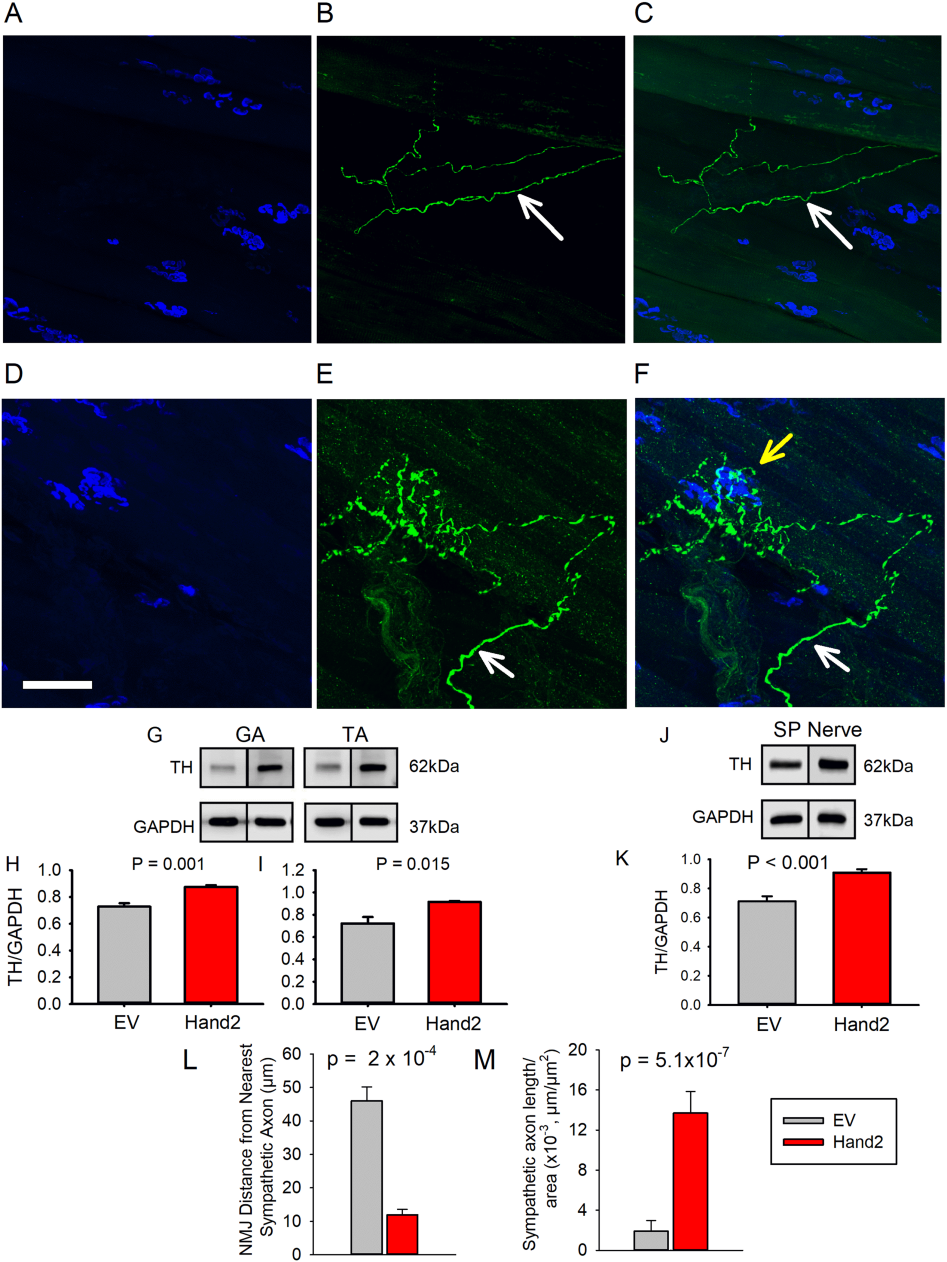
Sympathetic neuron heart and neural crest derivative 2 (Hand2) preserves the complexity of muscle sympathetic innervation and its proximity to the neuromuscular junction (NMJ). Confocal z‐stack confocal image of a whole‐mounted lumbricalis muscle from an EGFP‐treated *(A–C)* mouse and a Hand2‐treated *(D–F)* mouse. Post‐terminal *(A, D)*, TH+ axons *(B, E)*, and their overlay *(C, F)*. White arrows indicate sympathetic axons that do not establish contact with NMJs *(B–F)*, while the yellow arrow shows interaction between sympathetic axons and NMJs in a muscle from an old Hand2‐treated mouse *(F)*. Immunoblot analysis shows that Hand2 expression elevates TH expression in the gastrocnemius (GA) and tibialis anterior (TA) muscles *(G–I)* and the sciatic‐peroneal (SP) nerve *(J, K)* in old mice. *(L)* In the Hand2 mice, NMJ distance from the nearest sympathetic axon was shorter *(L)*, and the sympathetic axon path, normalized to the muscle cross‐sectional area, was longer *(M)*. *n* = 6 lumbricalis, SP nerves, GA, or TA muscles from six different mice per treatment group. Data were statistically analysed using a nonpaired *t*‐test.

*Figure* 6 shows NMJ post‐terminals (blue, A) and sympathetic axons surrounding blood vessels (arrows) (green, B) located at a considerable distance from the post‐terminals with no overlap (C). In contrast, Hand2‐treated mice show richer, more complex muscle sympathetic innervation than the EV group (*Figure* 6D–F). The white arrows show a sympathetic axon that overlaps with three NMJ post‐terminals (yellow arrow). Consistently, as determined by immunoblot (*Figure* 6G), TH levels are higher in GA (*Figure* 6H) and TA (*Figure* 6I) muscles as well as the sciatic‐peroneal nerve (*Figure* 6J, K). We also determined that Hand2 expression significantly decreased the distance between sympathetic terminals and NMJs (*Figure* 6L) while significantly increasing the density of sympathetic terminals per muscle area (*Figure* 6M). These results indicate that sustained Hand2 expression in SNs supports the complexity of muscle sympathetic innervation and its relationship with NMJs.

### Sustained sympathetic neuron heart and neural crest derivative 2 expression prevents activation of the myofiber Gα_i2_–Hdac4–myogenin–MyoD–atrogin/MuRF1 signalling cascade

*Figure* S6 shows that Hand2 maintains higher levels of the inhibitory G‐protein Gα_i2_ (A–C), which leads to lower Hdac4 (D–F), myogenin (G–I), MyoD (J–L), atrogin (M–O), and MuRF1 (P–R) levels, as tested by immunoblot in GA and TA muscles. These results indicate that Hand2 prevents activation of a signalling pathway previously demonstrated to be triggered by ablation of skeletal muscle sympathetic innervation.^21^

### Sympathetic heart and neural crest derivative 2 expression leads to increased PKA RI and Akt phosphorylation

*Figure* S7 shows more PKA RI (A, B), less PKA RII (C, D), a lower PKA/RII/PKA RI ratio (E), and significant Akt phosphorylation in both muscles from mice treated with Hand2 rather than EV (F, G). Although the GA muscle showed higher PKA RI levels, the difference was not significant, but the decrease in PKA RII and the lower PKA RII/RI ratio were significant in both muscles. These findings indicate that (i) chronic Hand2 treatment activates the canonical and non‐canonical β‐AR signalling and (ii) sympathetic Hand2 expression counters the age‐associated decrease in PKA RI, increases RII‐subunit levels, and lowers the PKA/RII/PKA RI ratio, which may influence AChR stability and myofiber innervation.

### Neuron heart and neural crest derivative 2 increases mTORC1 and FoXO1 phosphorylation but not NFκB in old mice

*Figure* S8A and B shows that Hand2 but not EV treatment increases mTORC1 phosphorylation levels at serine 2448, which is independent of Akt activity (see Discussion). However, both kinases might affect atrogin and MuRF1 levels and autophagy (see below). Also, FoXO1 (*Figure* S8C and D) but not FoXO3 (*Figure* S8E and F) phosphorylation increases with Hand2 treatment.

Decreased NFκB (*Figure* S8G, H) and IκB (*Figure* S8I, J) phosphorylation indicates that sustained skeletal muscle sympathetic innervation prevents NFκB–IκB complex dissociation and NFκB shift to the nucleus, preventing transcription of genes encoding proteins involved in inflammation signalling.

### Macroautophagy and chaperone‐mediated autophagy

*Figure* S9 shows that, compared with EV treatment, Hand2 treatment enhances p62 utilization (A, B) and the conversion of LC3‐I to LC3‐II (C, D) in GA and TA muscles. The increase in autophagy flux is associated with higher Atg7 levels in both muscles (*Figure* S9E, F). Analysis of changes in key components of the CMA pathway, such as lamp‐2A (*Figure* S9G, H) and heat shock cognate 70 kDa protein (*Figure* S9I, J) showed no significant differences between treatment groups.

### Sustained heart and neural crest derivative 2 sympathetic neuron expression in old mice regulates the skeletal muscle transcriptome

**FIGURE 7:**
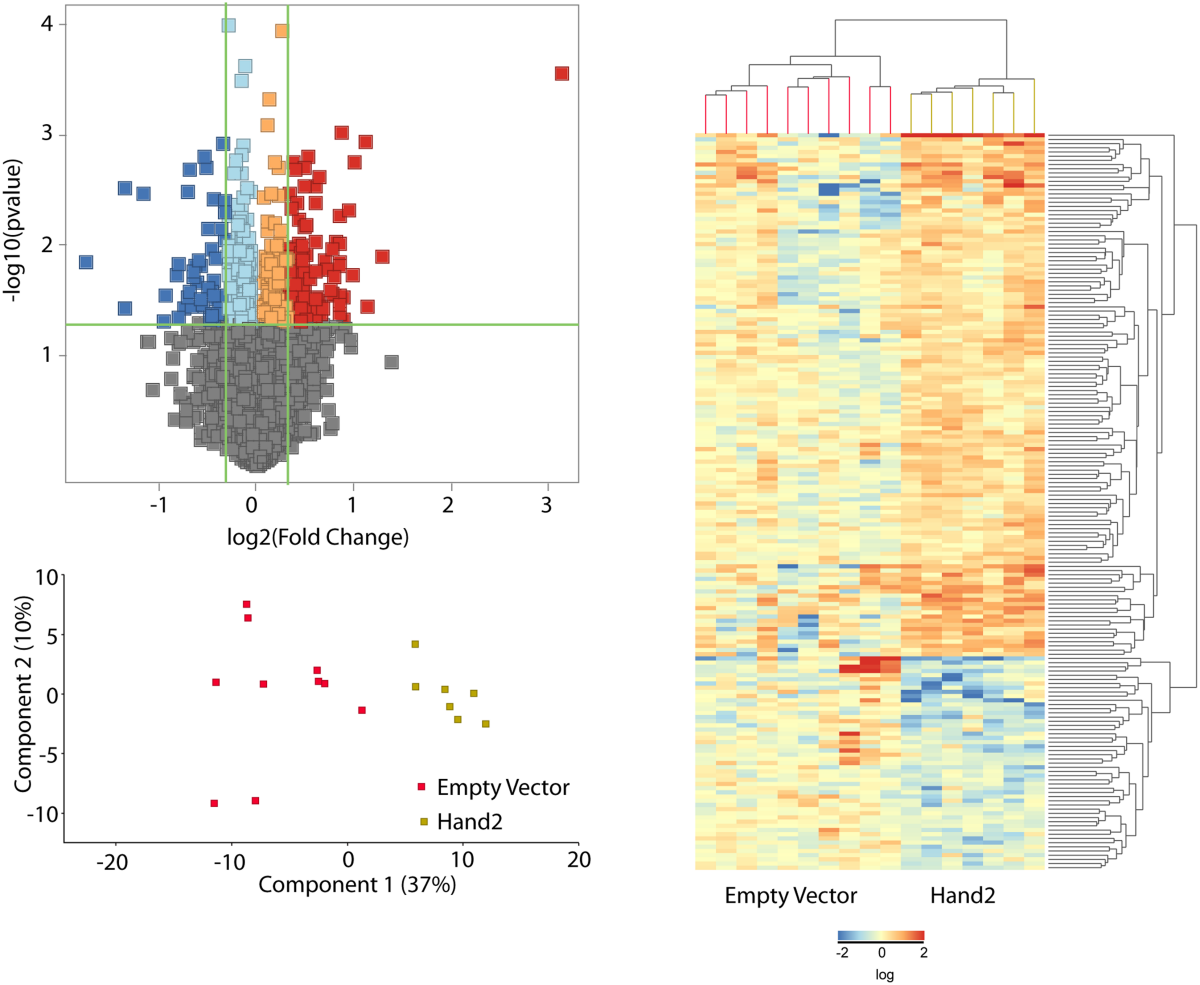
Sustained sympathetic neuron heart and neural crest derivative 2 (Hand2) expression regulates extensor digitorum longus (EDL) muscle gene transcription in old mice. *(A)* Volcano plot representing the significance (−Log10 *P*‐value) and magnitude of transcript change (Log2 fold change) with Hand2 treatment. *(B)* Principal components 1 and 2 plot for genes quantified for both treatment groups. *(C)* Heat map of 174 differentially expressed genes in the EDL muscle from empty vector (EV)‐treated (*n* = 10 muscles from 10 mice) and Hand2‐treated (*n* = 7 muscles from 7 mice) mice. Red and blue indicate up‐regulated and down‐regulated genes, respectively.

Previous work shows that SNS ablation extensively changes the skeletal muscle transcriptome.^21^ *Figure* 7A–C shows that Hand2 significantly regulates skeletal muscle genes, as quantified by RNA‐sequencing. Individual mouse values are listed in the Supplementary Database 3_ Hand2_EV Genes Differential Expression. Hand2 treatment seems to significantly up‐regulate rather than down‐regulate genes. However, the SNS seems to control the up‐regulation or down‐regulation of a combination of genes to reduce inflammation and to promote transcription factor activity, cell signalling, and synapse in the skeletal muscle.

Hand2‐induced *Tlr5* down‐regulation decreases IκB activation, leading NFκB to be excluded from nuclei and preventing activation of the canonical pro‐inflammatory pathway. Consistently, tumour necrosis factor‐like weak inducer of apoptosis (TWEAK) down‐regulation, up‐regulation of toll‐like receptor family‐1 (TLR‐1), and interleukin 16 contribute to the role of the SNS in inflammation and immune‐system function.

Hand2 treatment also increases expression of the gene that encodes *Ctrc3*, a member of the CREB‐regulated transcription co‐activator gene family, which regulates CREB‐dependent gene transcription in a phosphorylation‐independent manner and may select for cAMP‐responsive elements. Hand2 regulates the expression of genes involved in cell signalling, such as *Pde1a*, which regulates intracellular cyclic nucleotide concentrations through hydrolysis of cAMP and/or cGMP to their respective nucleoside 5′‐monophosphates, GDNF receptor (*Gfra1*), and many others, such as *Ucn*, *Caln1and Rcan1*, *Cpt1c*, and *Naip5*, which are involved in stress response, calcium signalling, beta‐oxidation, and apoptosis inhibition, respectively.^35^ Finally, Hand2 regulates genes encoding critical components of the NMJ and myofiber innervation, including *Nrcam*, a neural cell‐adhesion molecule,^3^ and *Nrxn1*, a presynaptic membrane cell‐adhesion molecule that primarily binds to neuroligins.^36^

## Discussion

**FIGURE 8:**
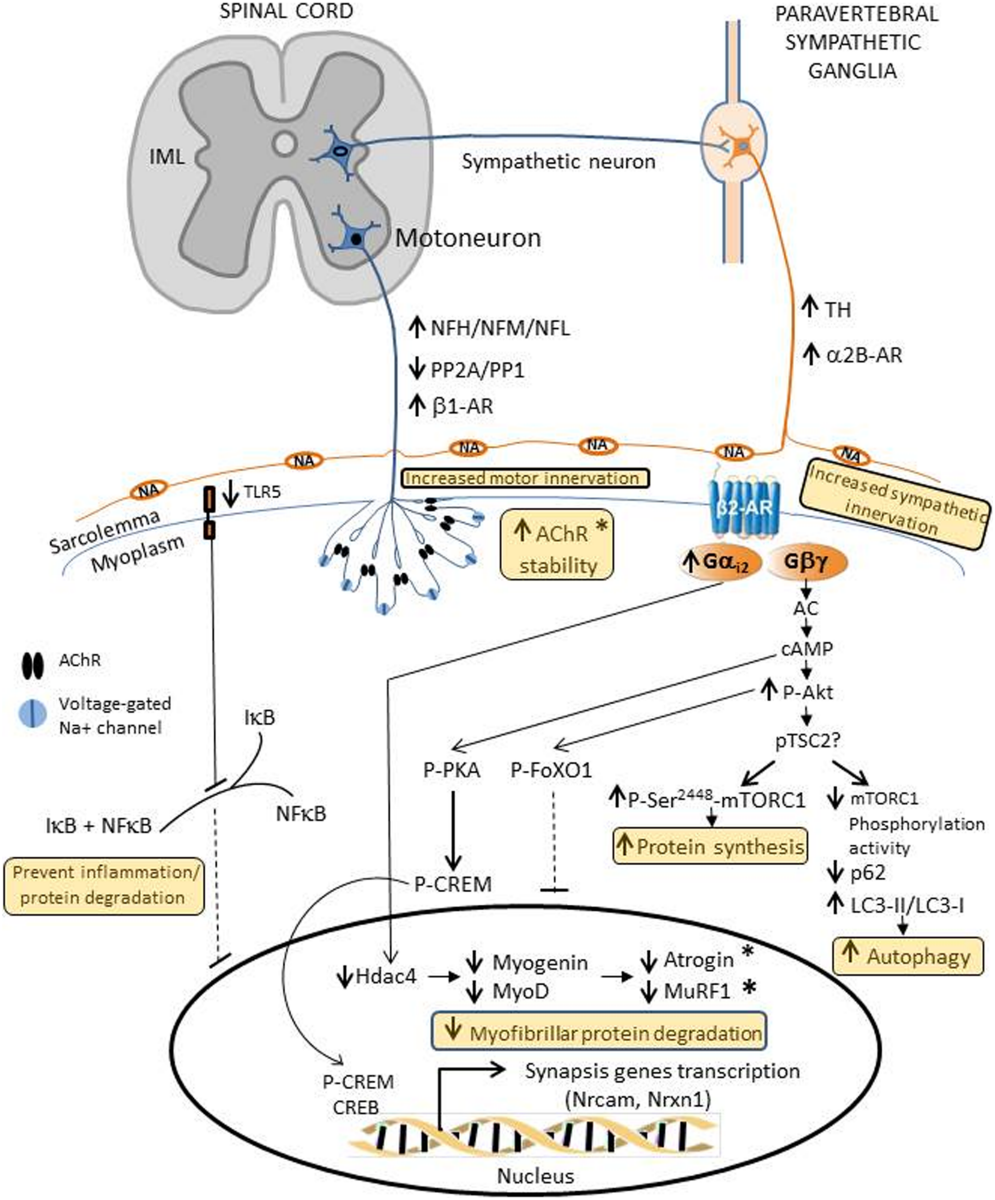
Schematic representation of heart and neural crest derivative 2 (Hand2)‐induced expression in sympathetic neurons and its impact on skeletal muscle sympathetic and motor innervation in old mice.

This study shows that selective expression of Hand2 in the mouse peripheral sympathetic system from middle age through old age increases muscle mass and force by (i) regulating skeletal muscle sympathetic and motor innervation; (ii) improving AChR stability and NMJ transmission; (iii) preventing inflammation and myofibrillar protein degradation; (iv) increasing autophagy; and (v) probably enhancing protein synthesis (*Figure* 8).

### Heart and neural crest derivative 2 expression declines throughout the mouse's life, but inducing its expression preserves the number and size of paravertebral sympathetic ganglion neurons even at advanced ages

Hand2 plays a prominent role in adult SNs' maintenance of NA genes^37^ and cell survival as demonstrated by post‐ganglionic sympathetic nerve axotomy experiments^38^ and conditional Hand2 KO in SNs.^30^ Adult nerve injury suppresses Hand2, which directly regulates dopamine‐hydroxylase (Dbh) expression and, most important, the genes controlling synaptic and neurotransmission functions in adult SNs.^30^ Based on its pivotal role, we focused our research on Hand2 expression with aging. As its expression is restricted to the peripheral SNS,^39^ we can rule out any influence of central sympathetic relays on our measures.

Neurons from 22‐month‐old EV‐treated mice were not immunoreactive to Hand2 Ab probably because they had <20% of the transcript recorded at P5. The reason for this decline is uncertain. SN hypermethylation may account, at least partially, for the age‐dependent decrease in *Hand2* gene transcription. Our data show CpG island hypermethylation in paravertebral sympathetic ganglion neurons with aging. Epigenetic silencing of Hand2 has been reported in the development of endometrial cancer, which occurs in older women.^40^ Hand2 undergoes age‐associated DNA hypermethylation in epithelial tissues.^41^ The mouse intestine, a tissue highly innervated by the SNS, shows a high rate of Hand2 hypermethylation as a function of age, suggesting that Hand2 can be hypermethylated in NA neurons from other territories as well.^41^

Compared with EV‐treated, Hand2‐treated mice have more ganglion SNs with larger soma size. This difference may be explained by age‐dependent neuronal depletion associated with less Hand2 expression.^30^ Conditional deletion of *Hand2* reveals its critical functions in neurogenesis during development^39^ and adulthood.^30^ Preserving the number and size of SNs in Hand2‐treated mice extends and enriches the complexity of sympathetic muscle innervation with aging (see below).

The increase in phosphorylated PKA RI and mTORC1 with Hand2 increases the phosphorylation of the *cAMP‐response element binding protein CREB modulator*—CREM, which translocates to the nucleus. Both CREB and CREM, bind a specific cis element—*cAMP‐response element* (CRE)—in the promoter region of skeletal muscle target genes.^42^ In the Hand2‐treated mice, up‐regulation of *Crtc3*, a member of the CREB‐regulated transcription co‐activator gene family, is consistent with activation of cAMP‐responsive genes. Our Ingenuity Pathway Analysis showed that CREM is a top upstream regulator of genes controlled by SNs expressing Hand2 in old age (*P*‐value = 3.17E−07). CREM targets include the *TH* and *dHand*, two essential genes for SN function and maintenance.^43^ In summary, reduced levels of PKA RI phosphorylation,^33^ together with genomic hypermethylation, may account for the age‐dependent decrease in *Hand2* gene transcription.

### Heart and neural crest derivative 2 increases skeletal muscle mass and force and whole‐body strength but not spontaneous mobility or endurance

The body weight of both EV‐treated and Hand2‐treated mice remained stable despite increased TA, GA, EDL, and soleus muscle mass, with no significant differences in visceral fat or spontaneous activity. Whether mice underwent loss in subcutaneous fat is uncertain at this point. Note that no heart hypertrophy was detected, which suggests that Hand2 expression in more cephalic levels of the sympathetic ganglion chain is less compromised than in the caudal regions innervating hindlimb muscles. This question remains.

The increase in muscle mass was associated with enhanced muscle strength. We attribute the observed increase in the Hand2 group to enlarged CSA in type II fibres in EDL muscle, and type I and type II fibres in soleus muscle.

Next, we tested force generation in response to electrical stimulation of nerves or muscles. For these experiments, we used lumbricalis muscle from mouse paws because it has (i) few myofiber layers, which favour oxygenation during prolonged stimulation recordings; (ii) banded alignment of its NMJs; and (iii) parallel arrangement of its tendons.^26^ Distal peroneal nerve stimulation resulted in unsustained and weaker peak force than that elicited by direct muscle stimulation in the EV‐treated compared with Hand2‐treated mice. However, this effect was more pronounced in response to muscle activation through the nerve. These data indicate that in addition to muscle sympathetic innervation, aging impairs motor innervation and NMJ transmission, but Hand2 expression in SNs rescues them significantly.

### Heart and neural crest derivative 2 expression enhances neuromuscular junction transmission and muscle innervation

We found that SN Hand2 enhances spontaneous and evoked NMJ transmission and attenuates myofiber sympathetic and motor denervation. Ablation of skeletal muscle sympathetic innervation alters NMJ transmission. Decreased MEPP amplitude and frequency, and decreased EPP amplitude and quantal content,^26^ are consistent with a decline in the compound muscle action potential and its rescue by sympathomimetics agents.^20^ Moreover, the Hand2‐associated increase in membrane and total AChR is consistent with our previous report that muscle sympathetic innervation regulates the receptor's stability.^26^ The enhanced expression of presynaptic AR reported here can explain how sustained muscle sympathetic innervation enhances NMJ transmission because SN regulation of motoneuron synaptic vesicle release, mediated by β1 and α2B‐AR, is blunted in geriatric mice.^26^, ^27^

Fragmentation and simplification of the post‐terminals are hallmarks of age‐dependent deterioration of skeletal muscle innervation, while sustained Hand2 expression by SNs preserves their molecular integrity and morphology. Hand2‐treated mice had fewer small post‐terminal fragments than the EV group and showed an approximately five‐fold decrease when the number of fragments was normalized to the muscle area. The wider range in post‐terminal fragment sizes in the Hand2 group indicates that, to a significant degree, muscle sympathetic innervation ameliorates fragmentation.

Skeletal muscle sympathetic innervation is better preserved in the Hand2 group than the EV group, as shown by (i) higher TH levels in nerve and muscle, detected by immunoblot; and (ii) the longer muscle sympathetic axon path and the shorter distance between SNs and NMJs, confirmed by immunohistochemistry. NMJ pre‐terminal and post‐terminal co‐localization and quantification of NF path length per muscle area show that preservation of sympathetic innervation enhances myofiber motor innervation. The mechanism underlying preservation of muscle sympathetic and motor innervation is uncertain; however, low PP2A and PP1 phosphatase levels and more NFH, NFM, and NFL subunit phosphorylation indicate that NF phosphorylation plays a role in stabilizing muscle motor innervation in Hand2‐treated mice. This conclusion is further supported by previous confocal and electron microscopy studies on muscle sympathetic ablation, which show compromised NF phosphorylation and disorganized axon NF and microtubules.^26^

Additionally, Hand2 up‐regulates the genes encoding critical components of NMJ and myofiber innervation, including *Nrcam*, a neural cell‐adhesion molecule and its product, NCAM, which is a marker of muscle denervation/reinnervation,^3^ and *Nrxn1*, a presynaptic membrane cell‐adhesion molecule that primarily binds to neuroligins, forming a complex required for efficient neurotransmission.^36^ Hand2 also up‐regulates the microtubule‐associated protein‐2 (*MAP 2*) gene, which may stabilize the microtubules against depolimerization, synapse differentiation inducing 1 like (*Sindig1l*), which plays a role in the shaping of synaptic specificity,^44^ and the GDNF receptor *Gfra1*, expressed at myelinated nerves and NMJ. Both GDNF and *Gfra1* play a crucial role in muscle innervation and GDNF signalling.^45^, ^46^

### Sustained sympathetic neuron heart and neural crest derivative 2 expression prevents activation of the myofiber Gα_i2_–Hdac4–myogenin–MyoD–atrogin/MuRF1 signalling cascade and PKA modifications associated with aging

The model of muscle sympathetic ablation shows down‐regulation of Gα_i2_, which activates the Hdac4–myogenin–MyoD cascade to raise MuRF1 levels and increase AChR destabilization.^26^ We found that Hand2 expression in SNs prevents decrease in Gα_i2_ levels with aging and averts this signalling cascade, which has been associated with muscle motor denervation.^47^ It lowers Hdac4 concentration to improve motor‐axon function^48^ and down‐regulates myogenin and myoD, both associated with muscle denervation, in GA and TA muscles. Note that down‐regulating this signalling cascade reduces atrogins. MuRF1 is expressed in both type I and type II fibres, but predominantly the latter; it is highly enriched in the post‐terminal; interacts with AChR in endocytic structures^49^; and participates in muscle trophism and maintenance.^50^ In sympathectomized MuRF1KO mice, AChR is partitioned between the sarcolemma and subsarcolemmal domain, as it is in innervated young mice.^21^ Consistently, Hand2‐induced preservation of muscle sympathetic innervation down‐regulates both MuRF1 and atrogin and prevents AChR endocytosis.

In old mice, the PKA RIα subunit is largely removed from—while PKA RIIα and RIIβ are enriched at—the NMJ.^33^ SN Hand2, like other interventions to counter muscle denervation,^33^ up‐regulates PKA RI in the TA muscle and down‐regulates PKA RII and the PKA RII/PKA RI ratio in both GA and TA muscles.

### Mechanisms that prevent age‐related decline in muscle mass and *ex vivo* force/*in vivo* strength

Hand2 treatment increases muscle Akt phosphorylation, a critical step in the activation of the non‐canonical β‐AR pathway, which leads to FoXO1 phosphorylation and exclusion from the nucleus and down‐regulation of MuRF1 and atrogin/MAFbx. This mechanism, together with down‐regulation of the Hdac4–myogenin–MyoD–atrogin/MuRF1 signalling cascade (see above), reduces atrogin expression to prevent muscle atrophy.^51^

Hand2 also decreased IκB and NFκB phosphorylation. NFκB signalling plays a role in age‐related skeletal muscle atrophy probably by activating the senescence‐associated secretory phenotype.^52^ These results support our RNA‐sequencing analysis showing that Hand2‐induced *Tlr5* down‐regulation decreases IκB activation, leading to nuclear exclusion of NFκB and preventing activation of the canonical pro‐inflammatory pathway. Consistently, TWEAK down‐regulation and TLR‐1 and interleukin 16, which play roles in the innate immune system and the cell cycle, respectively, contribute to SNS regulation of inflammation and immune‐system homeostasis.^53^ Cytokines down‐regulate *Hand2* in cultured SNs, while Hand2 overexpression rescues the noradrenergic phenotype. These observations support a third mechanism for decreased *Hand2* transcription with aging, in addition to reduced levels of PKA RI^33^ and Hand2 genomic DNA hypermethylation.

Rapamycin‐induced mTORC1 inhibition prevents age‐related muscle loss, while its chronic activation advances muscle damage and loss,^54^ inhibiting constitutive and starvation‐induced autophagy in skeletal muscle.^55^ We found that in both GA and TA muscles, Hand2 treatment increases mTORC1 phosphorylation at serine 2448, fine tuning its activity at the ‘repressor domain’. Deletion of this site strongly activates mTORC1.^56^ Increased phosphorylation at the repressor domain prevents ULK and ATG13 phosphorylation, activating autophagy.^57^

Does increased macroautophagy improve skeletal muscle mass and/or function in old mice? One common feature of all age‐related changes at the tissue level is the accumulation of damage and harmful modifications in DNA, proteins, lipids, and cellular organelles; autophagic efficiency declines over time, and intracellular waste products amass. Robust genetic evidence across species supports the inhibition of autophagy and aging.^58^ Analysis of muscle autophagy showed lower p62 and higher ATG‐7 levels and LC3‐I lipidation in the Hand2 group than the EV group. Atg7 ablation induces NMJ degeneration and precocious aging,^58^ which suggests that high ATG‐7 levels are required to improve muscle autophagy. Autophagy selectively degrades the LC3‐binding adaptor p62 protein (sequestosome‐1),^59^ and because a drop in p62 is associated with activation of the autophagic process, it serves as a readout of autophagic flux, together with LC3‐I conversion to LC3‐II.^60^ Non‐significant changes in Lamp2 and HSC70 levels indicate that macroautophagy, but not CMA, is activated by sustained Hand2 expression in SNs with aging. Note that direct stimulation of SNs activates muscle post‐synaptic β2‐AR, cAMP production, and import of the transcriptional co‐activator peroxisome proliferator‐activated receptor γ‐co‐activator 1α (PPARGC1A) into the myonucleus,^20^ which increases autophagy.^61^

In summary, the SNS regulates a number of primary synaptic and non‐synaptic events. Inducing Hand2 expression in SNs from middle‐aged mice improves their NMJ transmission, myofiber innervation, muscle mass, and function maintenance as they age.

## Author Contributions

OD conceived the study; OD and MLM designed the research; ACZR, MLM, ZMW, WMF, and HJB performed the experiments; OD, ACZR, MLM, LL, HJB, and WMF analysed the data, and all authors discussed the data; OD wrote the manuscript; WMF and ACZR contributed to writing the paper.

## Supporting information


**Data S1.** Supporting Information.
