# Supplementary material for: Heart and neural crest derivative 2‐induced preservation of sympathetic neurons attenuates sarcopenia with aging
Source: J Cachexia Sarcopenia Muscle. 2020 Nov 30;12(1):91–108. doi: 10.1002/jcsm.12644 (PMC7890150; doi:10.1002/jcsm.12644)
Supplement: Supplementary file 1 — Data S1. Supporting Information. [file JCSM-12-91-s001.zip › JCSM_12644_Supplementary Table 1_Abs.docx]

**Supplementary Table 1**

| **AntiboAntibody** | **Dilution** | **Source (Catalog No.)** |
| --- | --- | --- |
| α2B-AR | 1:250 | Alomone (AAR-021) |
| AChR | 1;1,000 | BioLegend (838301) |
| AkT-Phospho | 1:1,000 | Cell Signaling (3787) |
| AkT-Total | 1:1,000 | Cell Signaling (9272) |
| Atg7 | 1:2,000 | Sigma (A2856) |
| Atrogin | 1:1,000 | ECM Biosciences (AP2041) |
| β1-AR | 1:1,000 | Abcam (ab3442) |
| β2-AR | 1:1,000 | Abcam (13989) |
| FoxO1-Phospho | 1:1,000 | Cell Signaling (9461) |
| FoxO1-Total | 1:1,000 | Cell Signaling (2880) |
| FoxO3-Phospho | 1:1,000 | Cell Signaling (9466) |
| FoxO3-Total | 1:1,000 | Cell Signaling (2497) |
| Gα_i2_ | 1:200 | Santa Cruz (sc-13534) |
| GAPDH | 1:40,000 | GeneTex (GTX627408) |
| Hand2 | 15 µg/ml | R&D Systems |
| HDAC4 | 1:2,000 | Cell Signaling (7628) |
| HSC70 | 1:400 | Santa Cruz (sc-7298) |
| IK-Phospho | 1:500 | Santa Cruz (sc-8404) |
| IKB-Total | 1:400 | Santa Cruz (sc-371) |
| Lamp-2 | 1:500 | Abcam (13524) |
| LC3 | 1:1,000 | Cell Signaling (2775) |
| **Laminin** | **1:100** | **Millipore (MAB1914P)** |
| mTORC1-Phospho | 1:1,000 | Cell Signaling (2971) |
| mTORC1-Total | 1:1,000 | Cell Signaling (2983) |
| MuRF1 | 1:1,000 | R&D Systems (AF5366) |
| MyoD | 1:1,000 | BD Biosciences (554130) |
| Myogenin | 1:1,000 | DSHB (AB2146602) |
| NFH-Phospho | 1:1,000 | BioLegend (SMI-312) |
| **MHC-I** | **1:50** | **DBHS (Developmental Studies Hybridoma Bank-Univ Iowa (BA-F8)** |
| **MHC-IIa** | **1:500** | **DBHS (SC-71)** |
| **MHC-IIb** | **1:100** | **DBHS (BF-F3)** |
| NFH-Total | 1:1,000 | BioLegend (SMI-311) |
| NFкB-Phospho | 1:200 | Santa Cruz (sc-136548) |
| NFкB-Total | 1:200 | Santa Cruz (sc-8008) |
| NFL | 1:1,000 | Sigma (N5139) |
| NFM | 1:1,000 | Sigma (N5264) |
| p62 | 1:2,000 | Progen (GP62-C) |
| PKA RIα | 1:1,000 | BD Biosciences (610165) |
| PKA RIIα | 1:1,000 | BD Biosciences (612242) |
| PP1 | 1:1,000 | Thermo Scientific (MA5-17155) |
| PP2A | 1:1,000 | Millipore (05-421) |
| TH | 1:1,000 | Millipore (AB-152) |

**Antibodies Used for Immunoblot and Immunohistochemistry**
