# Supplementary material for: Heart and neural crest derivative 2‐induced preservation of sympathetic neurons attenuates sarcopenia with aging
Source: J Cachexia Sarcopenia Muscle. 2020 Nov 30;12(1):91–108. doi: 10.1002/jcsm.12644 (PMC7890150; doi:10.1002/jcsm.12644)
Supplement: Supplementary file 1 — Data S1. Supporting Information. [file JCSM-12-91-s001.zip › JCSM_12644_Supporting Information_text only.docx]

**Supporting Information**

**Hand2-Induced Preservation of Sympathetic Neurons Prevents Sarcopenia with Aging**

Anna Carolina Zaia Rodrigues,^1,2#^ Zhong-Min Wang,^1#^ María Laura Messi,^1#^

Henry Jacob Bonilla,^1^ Liang Liu,^3^ Willard M. Freeman,^5^ and Osvaldo Delbono^1-4§¥^

Department of Internal Medicine, Section on Gerontology and Geriatric Medicine;^1^ the Neuroscience Program;^2^ the Comprehensive Cancer Center;^3^ and the Sticht Center for Healthy Aging and Alzheimer’s Prevention,^4^ Wake Forest School of Medicine, Winston-Salem, North Carolina. Reynolds Oklahoma Center on Aging, Oklahoma City, Oklahoma^5^

**Supplementary Materials and Methods**

**RNA-seq and Data Analysis**

Library construction was performed in a stranded manner to retain transcripts’ directionality.[1] Each sequencing library was prepared from the RNA from a single animal to provide biological variance. Illumina Truseq Stranded HT library generation was performed according to manufacturer's instructions. Briefly, polyA containing mRNA was purified using oligo-dT attached magnetic beads. The mRNA was then chemically fragmented, and cDNA synthesized. For strand-specificity, the incorporation of dUTP instead of dTTP in the second strand cDNA synthesis does not allow amplification past this dUTP with the polymerase. After cDNA synthesis, each product underwent an end repair process, the addition of a single ‘A’ base, and finally ligation of adapters. To make the final library for sequencing, the cDNA products were further purified and enriched using PCR. Library sizing and quantification was performed by TapeStation (Agilent Technologies). Libraries were sequenced using an Illumina NextSeq (2x75bp) at the Oklahoma Medical Research Foundation Clinical Genomics Facility.

Reads alignment and filtering criteria included: adapter trimming, fixed 2bp trim from 5’ and 6bp from 3’ ends, a maximum number of one novel splice allowed per read, a minimum of 90% identity with the reference sequence, a maximum of 5% gap, trimming of 3’ end with Q<30. Alignment was performed directionally with Read 1 aligned in reverse and Read 2 in forward orientation. Normalization was performed with the DESeq algorithm [2]. Transcripts with an average read count value >10 in at least 100% of the samples in at least one group were considered expressed at a level sufficient for quantitation and those transcripts below this level were considered not detected/not expressed and excluded as these low levels of reads are close to background and are highly variable. For statistical analysis of differential expression, t-test followed by a fold change >|1.25| cutoff was used to eliminate those genes which were statistically significant but unlikely to be biologically significant and orthogonally confirmable due to their very small magnitude of change. Visualizations of hierarchical clustering and principle components analysis were performed in Strand Next Generation Analysis Software (NGS) (Version 3.3.1, Bangalore, India). The entirety of the sequencing data is available for download in FASTQ format from NCBI Sequence Read Archive. The GEO accession number is GSE151628.

**Real-Time PCR analysis**

Results were normalized to glyceraldehyde 3-phosphate dehydrogenase (*Gapdh*) RNA as the internal control. Relative quantities were calculated using the comparative threshold (*C*_T_) method.[3, 4] All samples were run in triplicate. Primers and Taqman probes were purchased from Applied Biosystems (Foster City, CA). Taqman primers for *Hand2* and *Gapdh* mRNAs analysis were Mm00439247_m1 and Mm99999915_gl (Applied Biosystems), respectively.

**Viral Vector Constructs**

To drive Hand2 transgene expression to NA neurons only, we used a specific NA neuron promoter and an adeno-associated virus (AAV) to build the AAV9-CAG-EGFP-CMV-PRSx8-Hand2-WPRE (pBK228) vector. AAVs do not exhibit pathogenicity in mice or humans and provide significantly longer transgene expression than other serotypes [5]. We selected an AAV-serotype 9 (AAV9) because it displayed the strongest expression of all AAVs in a comparative study of various serotypes carrying the same reporter gene. It is also rapidly cleared from blood after vein injection, and its expression is sustained for up to 12 months after the injection [5-7]. The capsid gene of AAV9 was pseudotyped by cloning into helper plasmids containing the AAV2 rep gene.

The packaging vector contained AAV2-ITRs (inverted terminal repeat sequences) and the PRSx8-driven *Hand2* transgene. The CAG promoter/enhancer was followed by EGFP and PRSx8 promoter consisting of tandem repeats of transcription factor Phox 2a/2b binding sites. PRSx8 promoter was followed by a transcription start site from the *hDBH* gene [8]. After PRSx8, the *Hand2* cDNA sequence (Genebank access: NM_021973.2), the Woodchuck Hepatitis Posttranscriptional Regulatory Element (WPRE) [9], and polyA+ were inserted. The total size of the expression cassette was 2.951 kb, below the AAV virus payload of 4.7kb. This hybrid transcomplementing construct, encoding replication genes from AAV2 and capsid genes from the AAV9 serotype. displays AAV9 cell tropism (henceforth, termed AAV9). The same AAV9, targeted to NA neurons and expressing EGFP but without the *Hand2* cDNA insert, was used as a control and termed empty vector (EV). The virus was produced by the triple-transfection method using HEK293 cells as described [[106](#_ENREF_106), [107](#_ENREF_107)].

**Viral Vector Delivery**

Viral vector (5 x 10^11^ vg of AAV9 in PBS supplemented with 5% sorbitol) was delivered by saphenous vein injection to isopentane-anesthetized, 16-month-old mice. The saphenous vein was exposed, and viruses injected with homemade sterile syringes coupled to a microelectrode. This approach ensured complete and safe IV delivery with minor leakage of the solution.

**Immnohistochemistry - *Skeletal Muscle Histochemical and Immunofluorescence Analyses***

Skeletal muscle histochemical and immunofluorescence analyses followed reported procedures.[10] Briefly, muscle samples were dissected, fixed in 4% paraformaldehyde (PFA), cryopreserved by sucrose gradient, embedded in Sakura Tissue Tek compound (Ted Pella, Redding, CA) at the optimum cutting temperature (O.C.T.), frozen in dry-ice‐chilled isopentane, and stored at −80 °C until cut into 10-µm sections at -20°C with a Leitz cryostat (Buffalo Grove, IL). Cryosections were rinsed in phospho-buffered saline (PBS) and blocked with 10% goat serum in PBS for 1 hour. Myofiber subtype characterization was based on identifying specific myosin heavy chain (MHC) isoforms. Muscle sections were incubated with primary antibodies against type-I, -IIa, and -IIb MHC (**see Supplementary Table 1**) and 10% goat serum in PBS for 2 hours at room temperature. After washing the sections with PBS for 5 minutes twice, they were incubated with the secondary antibodies AF 350 IgG2b (against BA-F8, 1: 500), AF 488 IgG (1SC-71, 1:500), and AF 555 IgM (BF-F3, 1:500) in 10% goat serum in PBS for 1 hour at room temperature followed by 2 more 5-minute washes with PBS. All secondary antibodies were purchased from Invitrogen, Thermo Fisher (Carlsbad, CA). Tissue sections were mounted using fluorescence mounting medium (S3023, Dako, Carpinteria, CA) and visualized with an inverted, motorized, fluorescent microscope (Olympus IX81, Tokyo, Japan) with an Orca-R2 Hamamatsu CCD camera (Hamamatsu, Japan). The camera driver and image acquisition were controlled with a MetaMorph Imaging System (Olympus).

Muscle fibers were identified as reported before[10], following a described procedure[11]. Immunofluorescence analysis on a single muscle cross-section using a cocktail of primary antibodies (BA-F8, SC-71, and BF-F3, against MHC-I, -IIa and -IIb, respectively) identified the four major adult fiber types, type-I (blue), type-IIa (green), type-IIb (red) and type-IIx (unstained) (**Supplementary Table 1**). This method also labeled hybrid fibers, which exhibit lower fluorescence intensity in both color channels relative to their respective pure fiber counterparts in a single muscle section [11] This approach was previously validated by staining serial sections with an antibody specific for type-IIx fibers (6H1, University of Iowa, Developmental Study Hybridoma Bank). Fibers immunoreactive to 6H1 antibody correspond to unstained fibers exposed to the antibody cocktail [11].

***Whole Mount Lumbricalis Muscle Immunohistochemistry and Confocal Microscopy Imaging***

We took advantage of the anatomical organization of the lumbricalis into few myofiber layers, which allowed us to fully image the intact muscle and assess muscle fiber innervation avoiding cryosection distortions [10]. Whole lumbricalis muscles were dissected and freed from surrounding tissues. The tendons were pinned in a dish coated with Sylgard 184 silicone (Dow Corning, Midland, MI), fixed in 2% PFA at 4°C overnight, washed in PBST three times, and blocked with 1% Triton and 10% goat serum in PBS at 4°C overnight. The next day, sympathetic terminals were immunostained for TH, nonphosphorylated or phosphorylated nerve neurofilament antibodies, synaptic vesicle protein 2 (**see Supplementary Table 1**), and α-bungarotoxin (BGT) CF680R (cat #00003, dilution: 1:250, Biotium, Fremont, CA) in 1% Triton and 4% goat serum in PBS at 4°C overnight. The third day, the preparation was washed 3 times in PBST. An AF568 goat anti-mouse IgG (cat #A11004, dilution: 1:1000, Thermo Fisher), used as the secondary antibody for SMI-311, SMI-312, and SV2, was added along with 1% goat serum at room temperature for 4h. NMJ postterminals were stained with tetramethylrhodamine 554 or AF680-BGT α-bungarotoxin (Molecular Devices, Sunnyvale, CA). The preparation was washed 3 times in PBS. Tendons were cut, and the lumbricalis muscle was mounted on a glass slide using Dako mounting medium and visualized with an Olympus FV1200/IX83 spectral laser scanning confocal microscope.

***Paravertebral Sympathetic Ganglia Immunohistochemistry and Confocal Imaging***

Whole-mount sympathetic ganglia were pinned in a dish coated with Sylgard, fixed in 2% PFA at 4°C overnight, mounted on a glass slide, rinsed in PBST, and blocked with 1% Triton and 10% goat serum in PBS at 4°C overnight. Ganglia were labeled with primary rabbit anti-mouse anti-TH polyclonal antibodies in the presence of 4% goat serum and 1% Triton in PBS at 4°C overnight. The next day, the preparation was washed in PBST; the secondary AF568 goat anti-rabbit IgG antibody (A-11036, dilution 1:1000, Thermo Fisher; Waltham, MA) was added; and the result incubated at 4°C overnight. The following day, the preparation was washed in PBST, and the nuclei were labeled with Hoechst 33342 (H3570, dilution 1:2000, Invitrogen, Carlsbad, CA) at room temperature for 5min. The preparation was then washed in PBS and mounted using mounting medium (S3023, Dako, Carpinteria, CA). A sympathetic chain in the mid-belly region of a ganglion was imaged on an inverted, motorized Olympus FV1200/IX83 spectral laser scanning confocal microscope. A third channel was used to acquire EGFP fluorescence. Image z-stacks were acquired with a UPLSAP020X objective, NA: 0.75. All ganglia were inspected in the z-dimension at 1.16 µm/slice resolution, and the step-size analysis avoided counting cells more than once. Total image size represented a 1,600 x 1,600 pixel area at 0.397 µm/pixel.

***Electrophysiological Recording of Neuromuscular Transmission***

The lumbricalis muscle was dissected with its plantar nerve attached as described [10, 12, 13]. The neuromuscular preparation was incubated in μ-conotoxin GIIIB (Alomone Labs, Jerusalem, Israel) to a final concentration of 1 µM for 30 minutes to prevent muscle contraction. The NMJ transmission was then recorded intracellularly in oxygenated normal mammalian Ringer’s solution (in mM, 135 NaCl, 5 KCl, 1 MgSO_4_, 15 NaHCO_3_, 1 Na_2_HPO_4_, 11 D-glucose, 2.5 calcium gluconate, pH 7.4) using a TEV-200A amplifier (Dagan Co., Minneapolis, MN), DigiData 1322A, and pClamp10.5 software (MDS Analytical Technologies, Sunnyvale, CA). The intracellular electrodes (~40mΩ) were filled with 2M K-citrate and 10 mM K-chloride and mounted on the stage of a MP-285 micromanipulator (Sutter Instruments, Novato, CA). An upright, fixed-stage Zeiss Axioscope FS microscope (Carl Zeiss, Thornwood, NY) with a 10 or 20X, water immersion FLUAR objective (Zeiss), mounted on a microscope translator (Sutter Instruments), was used to visualize the preparation. We recorded miniature endplate potentials (MEPPs) and evoked endplate potentials (EPPs) in an average of 15 NMJs per mouse. EPPs were elicited by electrical stimulation at increasing frequencies from 2 to 150 Hz. We used high-frequency stimulation (100-150 Hz) based on the reported frequency range for adult mouse spinal motoneuron firing [14]. MEPPs were baseline corrected, and their amplitude, frequency, time-to-peak, half-decay time, and duration were retrieved by setting basic selection criteria to detect peaks. Quantal content was calculated by dividing the mean amplitude of the EPPs by the mean amplitude of the MEPPs [15].

***Assessment of Muscle Force Generated by Direct Muscle- or Nerve-Evoked Stimulation***

We recorded nerve-evoked muscle contraction at increasing frequencies (2-150Hz) in EV- and Hand2-treated mice using an Aurora Scientific 407A force transducer and stimulator. We then blocked neuromuscular transmission with 10^-5^ g/ml d-tubocurarine. After verifying that nerve stimulation evoked no detectable muscle contraction, we switched to direct muscle-force recording using field stimulation generated between two parallel platinum plates and repeated maximal subtetanic and tetanic forces at the same frequencies in lumbricalis muscle. The composition of the bath solution was the same as that used for the NMJ transmission recordings. The experiment was carried out at room temperature (~21°C). Muscle force is expressed in millinewtons (mN). Due to the small mass of the lumbricalis muscle, we are unable to normalize muscle weights to body weights.

***Protein Isolation and Immunoblots***

***Total and membrane acetylcholine receptor pull-down and immunoblot.*** To isolate the total cytosolic AChR fraction, frozen muscles were mechanically disrupted using a mortar and pestle in liquid nitrogen and a handheld Tissue-Tearor^TM^ in ice-cold RIPA^®^ buffer (Sigma-Aldrich, St. Louis, MO) and a Roche complete Mini Protease Inhibitor Cocktail from Thermo Fisher Scientific. The resulting homogenate was centrifuged at 14,000 g for 5 min to remove insoluble debris, and the total amount of protein in the supernatant was measured.

BiotinXX-BGT (Thermo Fisher) was added to the proteins to start AChR pull-down, which was completed overnight with NeutroAvidin beads (Thermo Fisher). SDS-PAGE was conducted using a 4-20% gradient Mini-PROTEAN gel system (Bio-Rad Laboratories, Hemel Hempstead, Herts, UK). Gels were then transferred to 0.45 PVDF membranes (Amersham Health, Little Chalfont, Bucks, UK). For primary antibody incubation, blots were blocked in 5% nonfat dry milk with 0.1% TWEEN in tris-buffered saline (TBS: 150 mM NaCl, 50 mM Tris-HCl, pH 7.5). An anti-AChR α1 primary and anti-rat horseradish peroxidase (HRP) secondary antibody (Jackson Immunoresearch, West Grove, PA) were used. A monoclonal antibody (GeneTex, Irvine, CA) was used to normalize against GAPDH. Peroxidase activity was measured with either Chemiluminescent Substrate PICO or Maximum Sensitivity Substrate FEMPTO (Thermo Fisher), and band intensity was measured using a Licor C-DiGit Chemoluminescence Western Blot Scanner (Lincoln, NE). Band intensity was quantified with NIH ImageJ software.

To measure membrane AChR fraction, we injected increasing concentrations of bungarotoxin-biotin (2-4 µg) in different mice (weight: 20±0.5g) to determine the saturating dose (3µg), defined as that providing the maximal immunoblot signal. As expected, the saturating dose was close to the lethal dose (4µg) and was systematically injected into the left TA muscle. Mice were sacrificed 6h later, and the GA and TA muscles were dissected and processed as described above for AChR quantification.

To measure membrane AChR fraction, we initially injected increasing concentrations of bungarotoxin-biotin (2-4 µg) in different mice (weight: 20±0.5g) to determine the saturating dose (3µg), which was defined as that providing the maximal immunoblot signal. As expected, the saturating dose was close to the lethal dose (4µg) and was systematically injected into the left TA muscle. Mice were sacrificed 6h later, and the GA and TA muscles were dissected and processed as described above for AChR quantification.

***Protein measures in muscle lysates.*** For the remaining proteins analyzed in skeletal muscle lysates, we used the primary antibodies listed in **Supplementary** **Table S1.** Amersham HRP-conjugated secondary antibodies were purchased from GE Healthcare Life Sciences (Pittsburgh, PA).

Original selected muscle and nerve immunoblots from EV- and Hand2-treated mice are illustrated in **Supplementary Figure 10**.

**In-vivo Fatigue Recording**

In-vivo fatigue was recorded before and after treatment using a forced Exer 6 lane treadmill apparatus (Columbus Instruments, Columbus, OH) as described [16]. Mice were allowed to run on the treadmill for 5 min at 10 m min^−1^ on days 1 and 2 and 5 min at 10 m min^−1^ followed by 2 m min^−1^ increments until reaching 20 m min^−1^ for 2 min each period on day 3. The treadmill was not inclined. After training, mouse performance was recorded. The initial speed, 10 m min^−1^, reached a maximum of 5 min; we then increased the pace by 2 m min^−1^ every 2 min until exhaustion. We considered the mouse exhausted when it remained at least 10 s in the electric shock area of the treadmill. Maximal tolerated speed was recorded [17, 18].

**Inverted‐Cling Grip Test**

Grip strength was assessed using Kondziela's inverted screen test. This procedure provides a measure of overall strength and muscular endurance. The mice were allowed to acclimate in the experimental room for 10 min before testing, and the time they held from the net was computed. Results reflect the maximum time mice held to the net [18].

**Spontaneous Locomotor Activity**

Spontaneous locomotion in an open-field arena was assessed in both treatment groups. White noise was used to reduce potential anxiety, and illumination conditions were designed to maximize ambulation [19]. At the onset of the active, dark cycle (6-7 P.M.) under low-level, green LED illumination (room and arena ≤ 2 lux), a mouse was carefully transferred from its home cage to the polypropylene arena (46-cm x 61-cm floor, 50-cm walls), which was illuminated by undetectable 940-nm, far-infrared LED lights. Spontaneous locomotion was video recorded for 2 min using an infrared-sensitive CCTV camera (Panasonic WV-BP334) mounted 152 cm above the arena floor. After a 2-min trial, an object was added to the arena floor (prostrate resin cross; 8cm x 10cm area, 3cm high), and a 5-min trial was recorded. The arena was thoroughly cleaned after each trial. Recording and automated locomotor analysis were performed using Ethovision XT 7.0 (Noldus Information Technology, Inc.) with 3-point body tracking (nose, centerpoint, tail) at 29 frames/sec. Total distance traveled, average speed, maximal speed, and time spent in motion (start speed 13 cm/sec: stop speed 5 cm/sec) were calculated for each trial. Recording sessions of the 2- and 5-min trials were repeated over 3 consecutive days for each mouse [18].

**Supplementary Figure Legends**

**Supplementary Figure 1. Hand-2 treatment increases muscle weight and strength in aged mice.** Compared to EV-treatment, Hand2 significantly increased tibialis anterior (TA) (**A**), gastrocnemius (GA) (**B**), extensor digitorum longus (EDL) (**C**), and soleus (**D**) muscle, but not body (**E**), heart (**F**), or visceral fat (**G**) weight. Mobility, tested in the open arena setting, did not differ between groups in terms of spontaneous maximum speed (**H**), average speed (**I**), total traveled distance (**J**), time spent in motion (**K**), or treadmill running time (**M**), but net hanging time (**L**) was significantly longer in the Hand2- than EV-treated mice. We used 11 muscles from 11 EV-treated mice and 6-8 muscles from 6 Hand2-treated mice to count TA, GA, EDL, and soleus muscles and measure visceral fat; 10 hearts per group to measure heart weight; 11 and 7 mice per group to measure body weight; and 6 mice per group to assess spontaneous mobility and treadmill running. We used nonpaired t-tests for group comparisons except for treadmill data where we applied the ANOVA repeated measures test.

**Supplementary Figure 2.** **Sympathetic neuron Hand2 expression enhances NMJ transmission with aging.** Evoked end-plate potentials (EPPs) recorded at 2 (**A**), 10 (**B**), 25 (**C**), 30 (**D**), 50 (**E**), 100 (**F**), and 150Hz (**G**). Spontaneous miniature end-plate potential (MEPP) frequencies recorded in NMJs from EV- (left traces) or Hand2–treated (right traces) mice (**H**). **Table 1** quantifies NMJ transmission.

**Supplementary Figure 3. Sympathetic neuron Hand2 prevents age-dependent NMJ fragmentation.** A confocal image of whole-mounted lumbricalis muscles from EGFP- (**A**) and Hand2 (**B**)-treated mice. The NMJ postterminal was stained with BGT-680 (blue, cy5.5). Calibration bar = 50µm. The histograms for EGFP (**C**)- and Hand2 (**D**)-treated mice show the mean number of postterminals per area (0.1 µm^2^). Normalization of postterminal fragments per muscle area showed significantly fewer in the Hand2- than in the EV-group (**E**). For the analysis in **E**, we used 11 and 8 mice per group. Data were statistically analyzed using a nonpaired t-test.

**Supplementary Figure 4. Sympathetic neuron Hand2 enhances NF phosphorylation.** The Hand2 group has significantly more sciatic-peroneal (SP) nerve phospho-NFH (**A**), phospho-NFM (**B**), and phospho-NFL (**C**) than the EV group. Phosphatases PP2A (**D**) and PP1 (**E**) declined with sympathetic neuron Hand2 expression. Data were statistically analyzed using a nonpaired t-test.

**Supplementary Figure 5. Sustained sympathetic neuron Hand2 expression prevents activation of atrogenes.** GA and TA muscles Gα_i2_ (**A**), HDAC4 (**D**), myogenin (**G**), MyoD (**J**), atrogin (**M**), and MuRF1 (**P**) levels, analyzed by immunoblot. Digital optical density of the bands normalized to GAPDH is represented in the graphs **B-C**, **E-F**, **H-I**, **K-L**, **N-O**, and **Q-R**, respectively. N = 6 GA or TA muscles from 6 different mice per treatment group. Data were statistically analyzed using a nonpaired t-test.

**Supplementary Figure 6. Sustained sympathetic neuron Hand2 expression upregulates muscle PKA RI and increases AkT phosphorylation in old mice.** Hand2 treatment upregulated TA muscle PKA RI (**A, B**), but not PKA RII (**C, D**), and lowered the PKA RII/PKA RI ratio (**E**). Hand2 treatment increased phospho-Akt/total-Akt in both muscles (**F**), as quantified in **G**. N = 6 GA or TA muscles from 6 different mice per treatment group. Data were statistically analyzed using a nonpaired t-test.

**Supplementary Figure 7.** **Sympathetic neuron Hand2 regulates phosphorylation of key components of gene transcription, cell signaling, and inflammation.** Hand2 treatment increases mTORC1 phosphorylation (**A, B**) and FoXO1 (**C, D**), while decreasing FoXO3 in the TA (**E, F**) and NFκB (**G, H**) and IκB (**I, J**) phosphorylation in both GA and TA muscles. N = 6 GA or TA muscles from 6 different mice per treatment group. Data were statistically analyzed using a nonpaired t-test.

**Supplementary Figure 8. Sympathetic neuron Hand2 induces skeletal muscle macroautophagy flux, but not CMA.** Hand2-treated mice show less p62 (**A-B**), a higher LC3-II/LC3-I ratio (**C, D**), and more Atg7 (**E, F**) in both GA and TA muscles. Lamp2 (**G,** **H**) and HSC70 (**I, J**) levels did not differ significantly between groups. N = 6 GA or TA muscles from 6 different mice per treatment group. Data were statistically analyzed using a nonpaired t-test.

**Supplementary Figure 9.** **Increased Hand2 genomic methylation in aging ganglia SNs analyzed by bisulfite amplicon sequencing.** Methylation levels of mCH and mCHH in region 1 (57320498-57321005 chromosome 8) and region 2 (57321202-57322499 chromosome 8) were low, while mCG was higher in old (22-month) than in young (3-5-month) and middle-aged (13-month) C57BL6 mice (n = 24 ganglia chains from 12 mice equally divided in three age groups [young: 3-5, middle-aged: 13 months, and old; 22 months]). mCG: CG dinucleotide methylation, mCH, mCHH: any nucleotide methylation but G.

**Supplementary Figure 10.** Selected muscle and nerve immunoblots from EV- and Hand2-treated mice. Squares indicate the bands used for Figures’ illustration. Labels indicate the protein tested (top), the tissue used for the analysis (bottom), and the protein molecular size (left).

**Supplementary Table 1. Antibodies used for immunoblot and immunohistochemistry**

**Supplementary Database 1_Genomic DNA methylation Region 1.** Methylation levels of mCG, mCH, and mCHH in region 1 (57320498-57321005 chromosome 8). Data represent the mean of 6 paravertebral sympathetic ganglia from 3 mice.

**Supplementary Database 2_Genomic DNA methylation Region 2.** Methylation levels of mCG, CH, and mCHH in region 2 (57321202-57322499 chromosome 8). Data represent the mean of 6 paravertebral sympathetic ganglia from 3 mice.

**Supplementary Database 3_Hand2/EV Genes’ Differential Expression.** This spreadsheet shows RNA-seq data collected from 7 Hand2- and 10 EV-treated mice.

**References**

1. Sultan M, Dokel S, Amstislavskiy V, Wuttig D, Sultmann H, Lehrach H, et al. A simple strand-specific RNA-Seq library preparation protocol combining the Illumina TruSeq RNA and the dUTP methods. Biochem Biophys Res Commun. 2012;422:643-6.

2. Anders S, Huber W. Differential expression analysis for sequence count data. Genome Biol. 2010;11:R106.

3. Livak KJ, Schmittgen TD. Analysis of relative gene expression data using real-time quantitative PCR and the 2(-Delta Delta C(T)) Method. Methods. 2001;25:402-8.

4. Schmittgen TD, Livak KJ. Analyzing real-time PCR data by the comparative CT method. Nat Protocols. 2008;3:1101-8.

5. Zincarelli C, Soltys S, Rengo G, Rabinowitz JE. Analysis of AAV Serotypes 1-9 Mediated Gene Expression and Tropism in Mice After Systemic Injection. Mol Ther. 2008;16:1073-80.

6. Berns KI, Linden RM. The cryptic life style of adeno-associated virus. Bioessays. 1995;17:237-45.

7. Arimura S, Okada T, Tezuka T, Chiyo T, Kasahara Y, Yoshimura T, et al. Neuromuscular disease. DOK7 gene therapy benefits mouse models of diseases characterized by defects in the neuromuscular junction. Science. 2014;345:1505-8.

8. Hwang DY, Carlezon WA, Jr., Isacson O, Kim KS. A high-efficiency synthetic promoter that drives transgene expression selectively in noradrenergic neurons. Hum Gene Ther. 2001;12:1731-40.

9. Klein R, Ruttkowski B, Knapp E, Salmons B, Günzburg WH, Hohenadl C. WPRE-mediated enhancement of gene expression is promoter and cell line specific. Gene. 2006;372:153-61.

10. Rodrigues ACZ, Messi ML, Wang Z-M, Abba MC, Pereyra A, Birbrair A, et al. The Sympathetic Nervous System Regulates Skeletal Muscle Motor Innervation and Acetylcholine Receptor Stability Acta Physiol. 2018;225:e13195.

11. Bloemberg D, Quadrilatero J. Rapid determination of myosin heavy chain expression in rat, mouse, and human skeletal muscle using multicolor immunofluorescence analysis. PLoS One. 2012;7:e35273.

12. Rodrigues AZC, Wang Z-M, Messi ML, Delbono O. Sympathomimetics regulate neuromuscular junction transmission through TRPV1, P/Q- and N-type Ca2+ channels. Mol Cel Neurosci. 2019;95:59-70.

13. Wang Z-M, Rodrigues ACZ, Messi ML, Delbono O. Aging Blunts Sympathetic Neuron Regulation of Motoneuron Synaptic Vesicle Release Mediated by β1- and α2B-Adrenergic Receptors in Geriatric Mice. **J. Gerontol.** A **Biol. Sci. Med. Sci.** 2020;75 (8): 1473-1480.

14. Manuel M, Iglesias C, Donnet M, Leroy F, Heckman CJ, Zytnicki D. Fast Kinetics, High-Frequency Oscillations, and Subprimary Firing Range in Adult Mouse Spinal Motoneurons. J Neurosci. 2009;29:11246-56.

15. Sugiura Y, Chen F, Liu Y, Lin W. Electrophysiological Characterization of Neuromuscular Synaptic Dysfunction in Mice. In: Manfredi G, Kawamata H editors. Humana Press; 2011. pp. 391-400.

16. Pereyra AS, Wang ZM, Messi ML, Zhang T, Wu H, Register TC, et al. BDA-410 Treatment Reduces Body Weight and Fat Content by Enhancing Lipolysis in Sedentary Senescent Mice. J Gerontol A Biol Sci Med Sci. 2016;72:1045-53.

17. Messi ML, Clark HM, Prevette DM, Oppenheim RW, Delbono O. The lack of effect of specific overexpression of IGF-1 in the central nervous system or skeletal muscle on pathophysiology in the G93A SOD-1 mouse model of ALS. Exp Neurol. 2007;207:52-63.

18. Zhang T, Pereyra AS, Wang ZM, Birbrair A, Reisz JA, Files DC, et al. Calpain inhibition rescues troponin T3 fragmentation, increases Cav1.1, and enhances skeletal muscle force in aging sedentary mice. Aging Cell. 2016;15(3):488-98.

19. Valentinuzzi VS, Buxton OM, Chang AM, Scarbrough K, Ferrari EA, Takahashi JS, et al. Locomotor response to an open field during C57BL/6J active and inactive phases: differences dependent on conditions of illumination. Physiol Behav. 2000;69:269-75.
