# Supplementary figures and images for: Heart and neural crest derivative 2‐induced preservation of sympathetic neurons attenuates sarcopenia with aging
Source: J Cachexia Sarcopenia Muscle. 2020 Nov 30;12(1):91–108. doi: 10.1002/jcsm.12644 (PMC7890150; doi:10.1002/jcsm.12644)

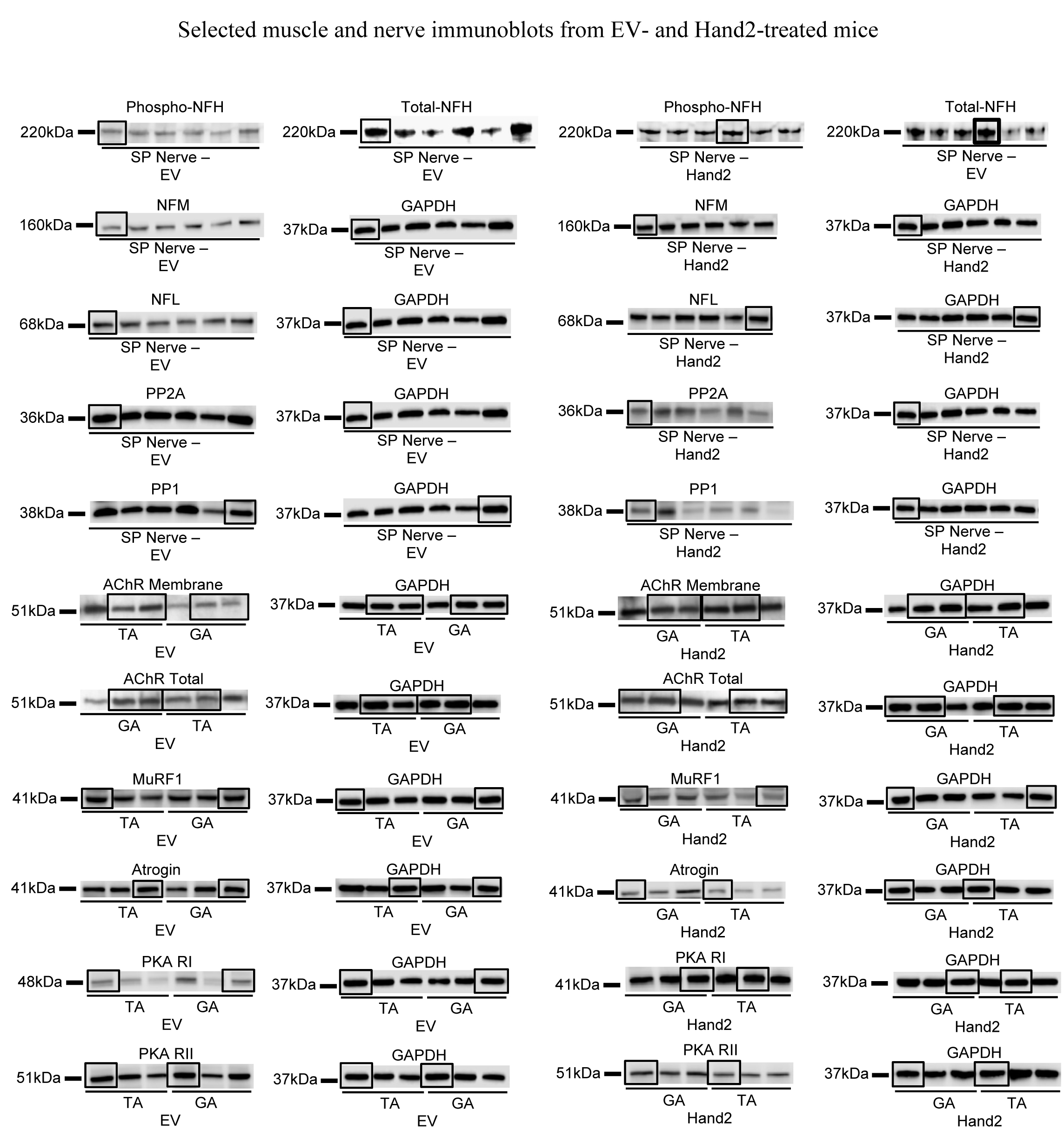

Supplement: Supplementary file 1 — Data S1. Supporting Information. [file JCSM-12-91-s001.zip › JCSM_12644_Supplementary Figure 10-1_crop.tif]

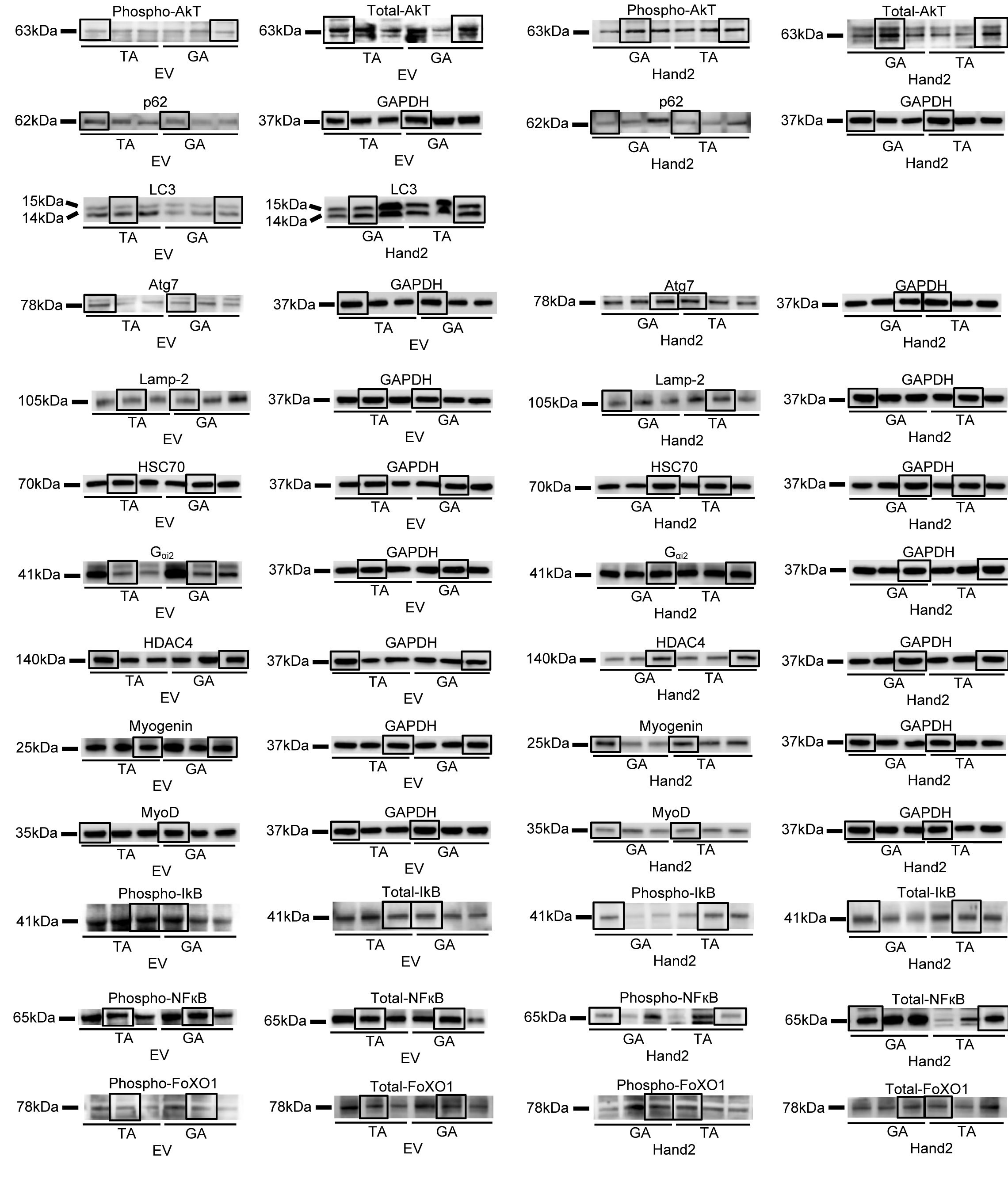

Supplement: Supplementary file 1 — Data S1. Supporting Information. [file JCSM-12-91-s001.zip › JCSM_12644_Supplementary Figure 10-2_crop.tif]

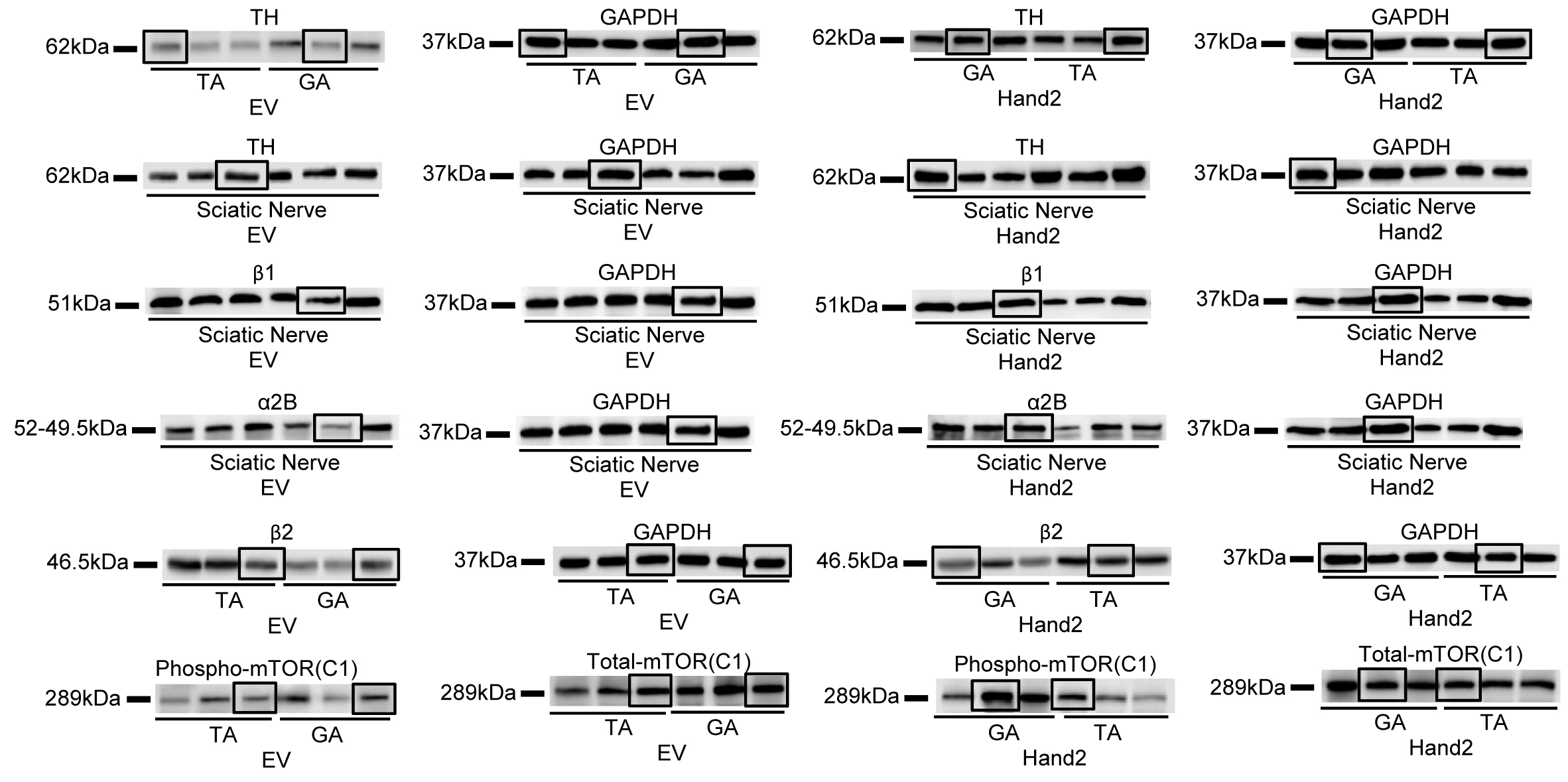

Supplement: Supplementary file 1 — Data S1. Supporting Information. [file JCSM-12-91-s001.zip › JCSM_12644_Supplementary Figure 10-3_crop.tif]
